# Face Masks in the New COVID-19 Normal: Materials, Testing, and Perspectives

**DOI:** 10.34133/2020/7286735

**Published:** 2020-08-07

**Authors:** Ming Hui Chua, Weiren Cheng, Shermin Simin Goh, Junhua Kong, Bing Li, Jason Y. C. Lim, Lu Mao, Suxi Wang, Kun Xue, Le Yang, Enyi Ye, Kangyi Zhang, Wun Chet Davy Cheong, Beng Hoon Tan, Zibiao Li, Ban Hock Tan, Xian Jun Loh

**Affiliations:** ^1^Institute of Materials Research and Engineering, Agency for Science, Technology and Research (A^∗^STAR), 2 Fusionopolis Way, Innovis, Singapore 138634; ^2^Department of Infectious Disease, Singapore General Hospital, Singapore

## Abstract

The increasing prevalence of infectious diseases in recent decades has posed a serious threat to public health. Routes of transmission differ, but the respiratory droplet or airborne route has the greatest potential to disrupt social intercourse, while being amenable to prevention by the humble face mask. Different types of masks give different levels of protection to the user. The ongoing COVID-19 pandemic has even resulted in a global shortage of face masks and the raw materials that go into them, driving individuals to self-produce masks from household items. At the same time, research has been accelerated towards improving the quality and performance of face masks, e.g., by introducing properties such as antimicrobial activity and superhydrophobicity. This review will cover mask-wearing from the public health perspective, the technical details of commercial and home-made masks, and recent advances in mask engineering, disinfection, and materials and discuss the sustainability of mask-wearing and mask production into the future.

## 1. Introduction

Emerging and reemerging infections have emerged as a threat to human health in recent decades [1]. Given how interconnected the world is today, a pathogen capable of human-to-human transmission can spark an outbreak far from where it originated. The virus causing the Middle East Respiratory Syndrome, for example, emerged in the Middle East but caused an outbreak in Korea. The world is in the midst of the COVID-19 pandemic, which is caused by the SARS-CoV-2 virus. Lockdowns and travel restrictions imposed to halt the spread of COVID-19 have led to devastating economic repercussions. The control of an infectious disease is based on knowledge of its mode of transmission. The recent COVID-19 pandemic is caused by the novel coronavirus, SARS-CoV-2, which is transmitted largely by the respiratory route (*vide infra*) [2, 3].

The best nonpharmaceutical interventions against disease spread via the respiratory route are broadly termed social or safe distancing measures, i.e., reducing close contact between individuals [4, 5]. Where safe distancing is not possible, personal protective equipment (PPE) is the accepted mode of self-protection. Masks and respirators are arguably the most important piece of PPE. They are a physical barrier to respiratory droplets that may enter through the nose and mouth and to the expulsion of mucosalivary droplets from infected individuals [6, 7]. Their role may be particularly important in COVID-19, where infected individuals may be shedding virus while asymptomatic or presymptomatic [8–10].

**Figure 1:**
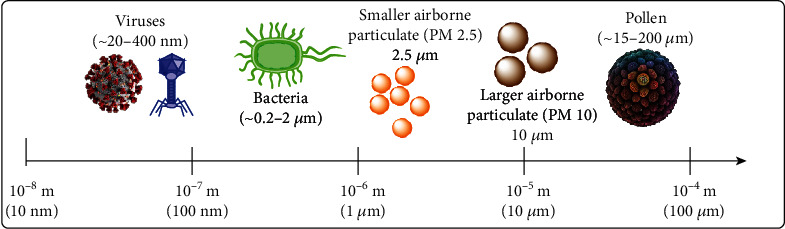
Relative size chart of common airborne contaminants and pathogens.

There are many different types of face masks and respirators offering different levels of protection to users [11–15]. Generally, masks do not fit tightly while respirators do. Masks and respirators may be reusable or disposable. Reusable ones include industrial-use half or full facepiece respirators with cartridge filters attached and homemade or commercial cloth masks; disposable ones include surgical masks, N95 respirators, and KN95 respirators. They all serve the general purpose of providing some form of protection against contaminants in the air, ranging from pollen to chemical fumes to pathogens. The filtering capacity, and hence the level of protection against pollutants and pathogens, depends on the materials used and the engineering design [11–15]. Contaminants in the air differ vastly in size (Figure 1). SARS-CoV-2 has a size ranging from 60 to 140 nm [16], smaller than bacteria, dust, and pollen. Therefore, masks and respirators made of materials with larger pore sizes, such as cotton and synthetic fabric, will not be able to effectively filter these viruses or tiny virus-laden droplets, as compared with those made of materials with much smaller pore sizes. Likewise, masks and respirators made of or coated with water-resistant materials are more effective against large virus-laden respiratory droplets and fluid spills. In addition to filtering capacity, factors such as user comfort and breathability also vary across different models. For instance, although the tight-fitting N95 respirator has filtering capacity superior to surgical masks, they have lower breathability and may cause discomfort after hours of wearing.

Mask-wearing can be effective in the containment of communicable diseases [17, 18] and has thus become a new normal in many societies in the COVID-19 pandemic. The surge in demand for surgical masks and respirators has led to a global shortage of supply and raw materials. As a result, many people have resorted to making their own masks, recycling used masks, or settling for masks offering less protection than actually needed. Researchers and industry players have therefore been working hard to address the issue of shortage, as well as to enhance the protection afforded by existing mask models. These efforts include (i) sourcing and engineering alternative materials with sufficient filtering capacity, (ii) engineering the design of masks and respirators for better protection, breathability, and user comfort, (iii) developing and engineering multifunctional masks and materials with hydrophobic, antimicrobial, self-disinfecting, and even sensing properties, and (iv) exploring new technologies for efficient production and customization of masks, e.g., 3D printing [19].

Attempts to enhance the mask will pivot on understanding the basics of mask technology. The fundamental questions, to our mind, are as follows: (i) how do masks (and the mask materials) protect us from pathogens; (ii) what are the existing models and materials of mask available in the market; (iii) how do they perform and how is their performance benchmarked against others; (iv) what are their limitations; (v) how can their performance be improved; (vi) what are some new features that can be incorporated into existing materials and models? This review seeks to address the above questions.

## 2. How Do Masks Protect Us against Airborne Diseases

### 2.1. The Respiratory Route of Transmission

A respiratory pathogen may be transmitted via three routes—contact, droplet, and airborne spread [20]. Contact transmission may be direct (i.e., transfer of virus via contaminated hands) or indirect (i.e., via fomites) [20]. Fomites are objects or materials that may carry infection, and spread by fomites means spread by touch. Viruses do survive for some time on inanimate objects, although the viral load declines dramatically [21]. If we touch a contaminated surface and then touch our eyes or nose, we may inoculate the virus into our mucosal surfaces. The role of touch in the spread of a respiratory virus is best exemplified by studies of the Respiratory Syncytial Virus (RSV) [22, 23]. The spread of SARS-CoV-2 via fomites has been elegantly demonstrated by real-world contact tracing, aided by closed-circuit cameras [24].

Droplet spread and airborne spread are different modes of transmission of the virus through the air. Viruses released when an infected person coughs, sneezes, sings, talks, or merely exhales may be found in particles of varying sizes [17]. Generally, particles larger than 5 *μ*m were thought to fall to the ground within 1 metre. More recently, however, the “gas cloud” hypothesis has been proposed [25]. Coughing, sneezing, or even exhaling produces mucosalivary droplets that exist as part of a cloud that “carries within it clusters of droplets with a continuum of droplet sizes” [25]. In combination with environmental factors, the “cloud” may be propelled up to 7–8 m. Wind speed, in particular, has been shown to play a role in determining the distance travelled by these particles [26].

Airborne spread occurs with pathogens found in exhaled droplets < 5 *μ*m in diameter. These particles remain afloat for some time and are able to travel long distances. Respiratory viruses accepted as being capable of spread via the airborne route include measles and varicella zoster (chickenpox). These viruses have a large *R*_0_, a feature thought to characterise spread by the airborne route. Interestingly, influenza, coronavirus, and rhinovirus RNA, generally thought to be transmitted by the droplet route, can be found in exhaled particles smaller or larger than 5 *μ*m [17, 27]. Further, viable influenza is present in particles smaller than 5 *μ*m. Hence, even viruses thought to be transmitted primarily by the respiratory droplet route may have the potential for airborne spread. Concern that SARS-CoV-2 may spread by the airborne route rose when it was shown to be viable for 3 hours in a drum that artificially kept particles afloat for several hours [21].

It might be less well known that more basic processes like talking can also lead to the release of potentially infectious droplets and aerosols. Using laser light scattering, it was found that there were average emissions of about 1000 droplet particles per second during speech, with high emission rates of up to 10,000 droplet particles per second [28]. By fitting the time-dependent decrease in particle detected to exponential decay times, the droplet particle sizes and estimated viral load could be calculated. The authors estimate that 1 min of loud speaking generates greater than 1000 droplets containing viruses [29]. Alternatively, respiratory particles of between 0.5 *μ*m and 5 *μ*m could be imaged by aerodynamic particle sizing. When participants made the “Aah” sound, there were emissions of up to 330 particles per second [30]. Taking into account that aerodynamic particle sizing measures particles under the detection limit of laser light scattering, these two methods can be seen to be complementary, and the total number of particles emitted could be even higher. In a separate study, droplet particle emission was shown to be directly proportional to loudness, with the number of particles emitted increasing from 6 particles per second when whispering to 53 particles per second at the loudest talking. The number of particles generated varied greatly across individuals, raising the possibility of superspreaders who could be the primary spreaders of viruses by talking [31].

### 2.2. Mechanistic Effect of Wearing a Mask

Masks and other PPE items serve as a physical barrier to respiratory droplets. With imaging using laser light scattering, it was found that the number of flashes, which corresponds to the number of respiratory droplets, could be kept at background levels by covering the speaker's mouth with a slightly damp washcloth [28]. An *in vitro* model with source and receiver mannequins was created to test the effect of the mask on filtering away radiolabelled aerosol emitted from the source. Masking at the source mannequin was consistently more effective at lowering radio-labelled aerosols reaching the receiver mannequin, whereas the only experimental setup where the receiver mannequin could be equally well protected was if the receiver mannequin wore an N95 mask sealed with Vaseline [32]. Therefore, masks can act as a physical barrier and seem to be more effective when worn by the droplet emitting person.

Masks have generally shown an effect in reducing virus emission from infected patients. The surgical mask was tested for its ability to block the release of various viruses by studying the amount of virus present in the exhaled breath of patients. The investigators were able to collect particles separated by size (> or <5 *μ*m). A significant drop in coronaviruses in both larger and smaller particles was observed with the mask on. The mask reduced influenza viruses found in larger but not smaller particles. After wearing a mask, no coronavirus was detected in all 11 patients, while influenza was detected in 1 patient's respiratory particles (out of 27). The mask did not lower rhinovirus counts in larger or smaller particles [17]. This suggests that surgical face masks can reduce the release of coronavirus and influenza from an infected person. In an earlier study for influenza, participants were induced to cough, and with both surgical masks and N95 masks, there was no influenza that could be detected by reverse transcriptase-polymerase chain reaction (RT-PCR) for 9 infected patients [33]. When the exhaled influenza virus was separated into the fractions based on size, it was found that surgical masks were highly effective at removing influenza from the larger coarse fraction (≥5 *μ*m) but less effective from the fraction with smaller particles [34].

Wearing masks has also been shown to protect individuals coming into contact with an infected person. In a survey of 5 hospitals in Hong Kong during SARS, hospital staff were asked about the protective measures they took and this information was correlated with whether they were infected by SARS. It was found that wearing masks was the single most important protective measure in reducing the chance of getting infected (*p* = 0.0001), and the people who wore either surgical masks or N95 masks were not among the 11 infected staff. There were however 2 instances of people who wore paper masks being infected, suggesting that the type of masks was also important [35]. A study compared the effectiveness of N95 and surgical face masks against viral respiratory infections in healthcare workers. Healthcare workers had no significant difference in influenza infection outcomes when wearing N95 and surgical masks, suggesting that both types of medical masks could protect similarly [36]. A meta-analysis was performed on clinical studies to explore the protective effect of masks. The risk ratio was calculated for the incidence of infection in the protected group vs. the unprotected group, where risk ratio < 1 suggests a reduced risk. Wearing a mask protected individuals against influenza-like illness, showing a risk ratio of 0.34, with a 95% confidence interval between 0.14 and 0.82. Similar to the study above, surgical masks and N95 masks showed little difference in protection, with a risk ratio of 0.84 and a 95% confidence interval of 0.36-1.99 suggesting no significant difference in risk [37].

**Figure 2:**
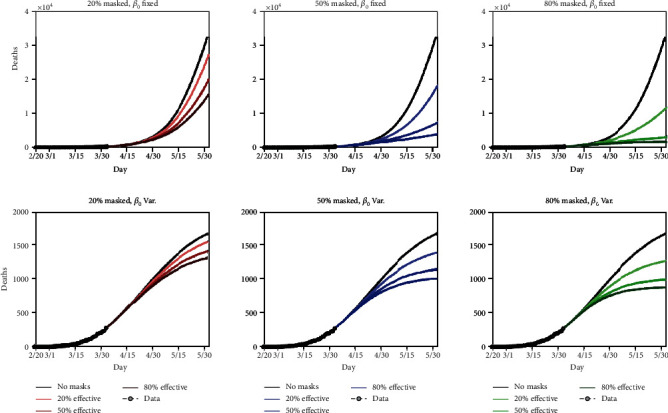
Simulated future (cumulative) death tolls for Washington state, using either a fixed (top panels) or variable (bottom panels) transmission rate, *β*, and nine different permutations of general public mask coverage and effectiveness. The *y*-axes are scaled differently in the top and bottom panels. Reproduced with permission from Ref. [18]. Copyright 2020, Elsevier B.V. on behalf of KeAi Communications Co., Ltd.

Recently, a modelling study performed by Eikenberry et al. based on COVID-19 infection data obtained in New York and Washington suggested that the broad adoption of face mask by the general public can significantly reduce community transmission rate and death toll [18]. As shown in Figure 2, based on data obtained from 20^th^ February to 30^th^ March, the cumulative death rate was projected to be reduced to a greater extent as more people wear masks over the next 2 months. Therefore, the study concludes that community-wide adoption of face mask has great potential to help curtail community transmission and the burden of the COVID-19 pandemic.

### 2.3. Advantages and Caveats of Wearing Masks

Mask usage, in addition to other nonpharmaceutical interventions, can be an effective containment measure in an epidemic. Face masks can prevent dispersal of droplets when infected persons talk, sing, cough, or sneeze. The rate of emission of particles correlates with voice loudness during speech or other vocal activities [38]. A physical obstruction that prevents the wearer from touching the face, a mask may lead to better hand hygiene [24]. The reverse is also true—an increased tendency for wearers to touch their faces, such as when adjusting their masks [39]. Even with the right mask, wearers can still be infected if droplets enter via the eyes, thus highlighting the importance of additional protection [40].

Masks also reduce the risk of environmental contamination by respiratory droplets [24]. As mentioned, SARS-CoV-2 transmission via fomites has been documented [24]. In reality, usage by each individual varies. The mask may not fully cover the mouth and nose, or it may be used and reused too frequently. These can mean huge variations in mask performance outcomes [24, 41]. In addition, wearers should avoid touching their faces and the external surface of their masks. Hand hygiene also varies from person to person. Hence, mask usage must be complemented by other behavioral changes for effective infection prevention. Finally, the universal use of face masks prevents discrimination of individuals who wear masks when unwell because everybody is wearing a mask. Universal mask-wearing can create new social norms, motivating individuals to wear masks at the initial onset of symptoms without fear of being stigmatized. The unintentional infection of healthy individuals by asymptomatic and presymptomatic persons can be avoided [39, 40]. Masks are visible indicators of crisis mode, which can prompt behavioral changes such as social distancing and frequent handwashing [39].

### 2.4. Complications of Asymptomatic and Presymptomatic Transmission

In the early stages of the COVID-19 pandemic, many countries did not recommend mask-wearing by healthy people to prevent panic buying and stockpiling [41]. Such paranoia can lead to a drastic shortage of medical masks for healthcare workers. The efficacy of masks in protecting individuals from being infected was also doubted. Since then, governments, such as Singapore's, have made it mandatory to wear a mask in public [42]. Such a change of policy direction has come about primarily because of increasing recognition of the concept of asymptomatic and presymptomatic transmission.

There has been a rise in asymptomatic and presymptomatic cases reported in many parts of the world. In an early example of such transmission, five symptomatic patients contracted the virus from one asymptomatic relative who travelled from Wuhan to Anyang [43]. In the same time period, another family cluster of three travelling from Wuhan to Guangzhou revealed asymptomatic transmission [44]. While the adult male presented clinical symptoms, his wife and son were both asymptomatic. All three tested positive for COVID-19 on RT-PCR. A separate study on 82 residents at a long-term care skilled nursing facility revealed that out of the 30.3% positive cases, 43.5% were symptomatic while more than half were asymptomatic [45]. After 1 week, this asymptomatic group was reassessed and 10 out of 13 developed symptoms, leading to their reclassification as presymptomatic. Likewise in Singapore, 243 cases comprising of seven clusters could be explained by presymptomatic transmission [38]. In four of these clusters, the exact date of transmission could be determined to lead to the conclusion that transmission occurred 1-3 days before symptoms appear in the source patient. These cases demonstrate that viral shedding can occur prior to the onset and absence of symptoms, thus complicating the containment of this COVID-19 pandemic. Various governments have to enforce social distancing, good hygiene practices, and mask usage to effectively contain asymptomatic and presymptomatic transmission.

### 2.5. Household Mask Usage

It is a lot more ambiguous when it comes to household mask usage. What we do know is that wearing a mask or protective covering can reduce the emission of droplets and infectious viruses from the infectious person [17, 32, 40]. Laser light scattering studies revealed that covering the mouth of a speaker with damp cloth reduced particles emitted to background levels. Therefore, wearing a cloth mask or even a scarf, as recommended by the Centers for Disease Control and Prevention (CDC), to cover the nose and mouth would serve to reduce respiratory emissions from an infected person, whether he is symptomatic or asymptomatic [46].

The type of mask material worn is important in mitigating the risk of infection. For instance, a study found that in a healthcare setting, the risk of influenza was substantially higher in the cloth mask group than the medical mask group [47]. Hence currently, surgical face masks and N95 respirators are still the best option, if available, for protecting a healthy person in a high-risk environment [35]. A more comprehensive experimental investigation on the protective effect of reusable cloth masks is urgently required, particularly during an extended pandemic period when a sustainable low-cost option is essential for household usage.

Studies on the effect of wearing face masks in households have been plagued by confounding factors and adherence issues. While face masks and hand hygiene have been known to be key protective measures against droplet and fomite transmission, a study of 259 households in Hong Kong showed no significant difference in the infection risk for the group that both wore face masks and observed hand hygiene [48]. A similar conclusion was reached among young adults living in university residence halls, where a combination of face masks and hand hygiene did not correlate with a significant decrease in the rate of influenza-like illness [49]. A separate study provided some insight into the underlying reason. While there was no significant decrease in infection risk by wearing face masks, it was found by self-reporting that less than 50% of study participants wore masks most of the time. In the group that wore masks most of the time, it was found that the risk of infection decreased, with a risk ratio of 0.26, with a 95% confidence interval of 0.06 to 0.77 [50]. Face mask-wearing in households seems to be ineffective for seasonal infectious diseases. Whether or not mask-wearing at home should be regulated or recommended is doubtful, since household members will necessarily eat together, an activity during which masking is impossible. Normalcy at home is critical to mental health in a pandemic.

### 2.6. Public Policy and Population-Level Effects

As discussed, asymptomatic and presymptomatic cases have made pandemic containment increasingly challenging, resulting in a paradigm shift in government approaches. Undocumented cases, many of whom were asymptomatic or mildly symptomatic, were possibly contributing to a large number of infections (79%) in China [51]. A random sampling of 3000 New York residents at various locations, such as grocery stores, revealed an infection rate of 13.9%, and an estimated 2.7 million people might have been infected [52]. On 6th April, an interim guideline from the WHO stated that healthy people did not need to wear masks because there was no evidence that masks can protect the wearers [53]. This contrasted with CDC guidelines on 3rd April, which recommended cloth face coverings in public spaces, especially where there is significant community-based transmission [46].

Anecdotal evidence within hospital settings showed that universal mask usage must be implemented in high-risk areas. Symptoms of COVID-19 are similar to other respiratory diseases, and some healthcare workers displaying mild symptoms continue to work [39]. In another multiyear single-center study, a mask-wearing policy was instituted for all who interacted with hematopoietic stem cell transplant patients. Comparing the mask and premask years, respiratory viral infections decreased significantly after the mask policy. Hospital-wide and in an adjacent hematologic malignancy unit, the absence of mask policy meant that infections remained high [54].

A 2003 SARS study on five Hong Kong hospitals revealed that staff who adopted all four measures of masks, gloves, gowns, and handwashing remained healthy. Staff who omitted at least one of these practices became infected but the wearing of masks was the most significant and important measure [35]. The other three measures conferred no additional significant protection to mask wearers. Hence, stopping droplet transmission at the face level is critical.

Mathematical modelling on the 2009 (H1N1) influenza concluded that if masks were enforced early at 100 versus 1000 infectious people, the severity of an outbreak could be reduced markedly [55]. Everyone, not only infectious individuals, must wear masks to significantly reduce the cumulative number of cases. In this model, the effectiveness of surgical masks was low and insignificant. For N95 respirators operating at 20% effectiveness, a significant reduction of influenza (20%) was achieved if only 10% of the population wore them. If 25% and 50% of the population complied, the reduction became 30% and 36%, respectively.

For COVID-19, similar conclusions were achieved in a theoretical model and empirical data set study [56]. Monte Carlo simulation and an SEIR (susceptible-exposed-infectious-recovered) model were used. When a minimum of 80% of people wore masks, the impact on the pandemic was significant and the curve flattened. However, this intervention failed when 50% or less of the population wore masks. By day 50 of a regional outbreak at the latest, universal masking could prevent widespread transmission. If enforced at day 75 with a 90% masking adoption, there was no impact on the spread of infection, highlighting the importance of early masking intervention. If at least 80% of the population wore masks, the curve could flatten more significantly than enforcing a strict lockdown. In addition, after lockdown, allowing social distancing without masking led to uncontrollable rise in infections.

With the results of these two simulations in mind, the authors studied the impact of masking enforcement on infection growth across many countries [56]. Countries can be classified into three tiers from best- to worst-performing. Across the tiers, the average daily infection growth rate and reduction from the peak are as follows: top at 5.9% and 74.6%, middle at 14.2% and 45.8%, and low at 17.2% and 37.4%. Best-performing countries instituted universal masking orders before 15th March while those in the middle tier did this after the date. The remaining countries were the worst-performing. Overall in every region, employing universal masking resulted in better management of COVID-19.

Even though the Hong Kong government recommended mask usage only for symptomatic people, as with WHO guidelines, the general public volunteered to wear mask proactively [24]. Universal masking was also advocated by leading experts in clinical microbiology and infectious disease specialties. After 100 days, the number of infected per million population in Hong Kong was significantly lower than countries without universal masking. The comparison was done for countries with similar population density, healthcare system, BCG vaccination, and social distancing measures. These countries include Singapore, South Korea, Spain, Italy, and Germany. While infections and deaths have skyrocketed worldwide, Hong Kong has recorded low numbers, particularly remarkable given its close proximity to China and high population density. The main difference causing the favourable outcome in Hong Kong was the voluntary universal masking among residents since early in the pandemic. By observation of morning commute over three consecutive days, only 3.4% out of 10,050 persons failed to wear masks. There were eleven COVID-19 clusters which could be attributed to recreational mask-off settings such as dining and drinking in restaurants or bars, singing at karaoke parlours, and exercising in gymnasiums.

Therefore, these experiments and population studies show that universal masking is effective if implemented early and rigorously. Governments should deploy resources to obtain sufficient masks in order to achieve sustainable universal masking. If supplies are insufficient, the general public should use cloth masks when they are outside their homes. Medical masks should be reserved for healthcare workers and others who perform essential functions [56].

## 3. Understanding Performance of Commercial Mask

### 3.1. 3-Ply Surgical Mask

**Figure 3:**
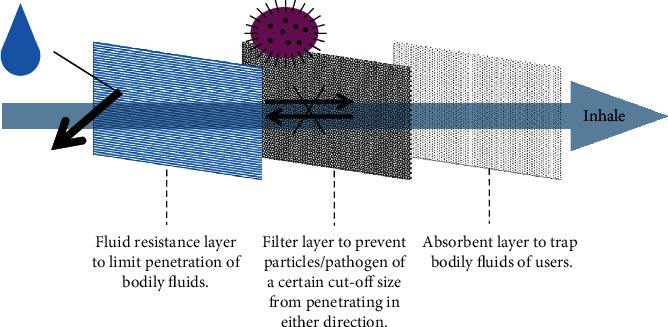
Illustration showing the function of each individual layers of a 3-ply surgical mask.

The 3-ply surgical mask is commonly used in the COVID-19 pandemic. The 3-ply surgical mask is made up of 3 different layers of nonwoven fabric with each layer having a specific function, as shown in Figure 3. The outermost layer (typically blue) is waterproof and helps to repel fluids such as mucosalivary droplets. The middle piece is the filter, which prevents particles or pathogens above a certain size from penetrating in either direction. The innermost layer is made of absorbent materials to trap mucosalivary droplets from the user. This layer also absorbs the moisture from exhaled air, thus improving comfort. Together, these 3 layers effectively protect both the user and the surrounding people by limiting the penetration of particles and pathogens in both directions.

As suggested by its name, nonwoven fabric does not contain intertwining strands and is made by bonding a mass of fibres together using heat, chemical, or mechanical means. Felt is one of the most common examples of nonwoven fabric. Although nonwoven fabric is mechanically weaker than its counterpart, it is cheap and fast to manufacture. Therefore, it is an ideal material for the surgical mask. The two most common methods of making nonwoven fabric for surgical mask are spunbond and melt-blown.

**Figure 4:**
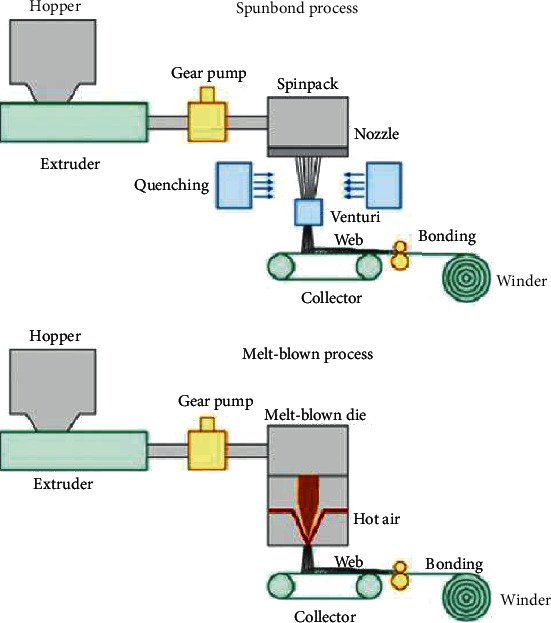
Schematic illustration of spunbond and melt-blown process. Republished with permission from Ref. [59]. Copyright 2015, Butterworth-Heinemann.

The spunbond process combines the spinning and sheet formation process into one continuous, nonwoven manufacturing system [57, 58]. As seen in Figure 4, the spunbond process consists of several integrated steps, namely, extruder, gear pump, spinpack, quencher, collector, bonder, and winder.

- Extrusion is the process where the polymer is melted by heat and mechanical action of the screw
- The gear pump plays a critical role in controlling the precise volumetric flow rate of the molten polymer. This is a key step to maintain a uniform temperature of the molten polymer
- The spinpack is a die block assembly which turns the molten polymer into uniform thin filaments and is designed to be able to withstand 300°C to 400°C
- The filaments are then quenched by cool air
- After quenching, the filaments are collected together as filament web on a moving belt
- The filaments in the web are then bonded together via heat, chemical, or mechanical means to form the nonwoven fabric
- Lastly, the nonwoven fabric is collected in the winder

Although the melt-blown process is very similar to the spunbond as seen in Figure 4, the microfibres produced in melt-blown are much finer and the pore size of the nonwoven fabric can be much smaller. Therefore, due to the finer pore size, melt-blowing is the typical process used to fabricate the middle filtering piece of the 3-ply surgical mask. The melt-blown process also consists of several integrated steps, namely, extrusion, gear pump, die assembly, collector, and winder [60]. The major difference between spunbond and melt-blown is in the die process which is the most important element responsible for the smaller diameter microfibres. There are three components in the die assembly: the feed distribution plate, die nosepiece, and air manifold which are all kept heated at 215°C to 340°C.

**Figure 5:**
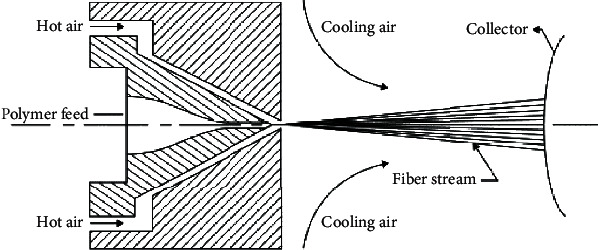
Schematic illustration of air manifold in melt-blown process. Republished with permission from Ref. [61]. Copyright 2014, Woodhead Publishing Limited.

- The feed distribution plate ensures the molten polymer flows across the plate evenly. The shape of the feed distribution plays an important role in the polymer distribution. The most common, coat hanger-type, has a manifold at the polymer entrance to ensure even and uniform distribution of polymer flow
- The die nosepiece is the key component which ensures the filament diameter and quality. The die tip is a very wide and thin metal piece with orifice measuring about 0.4 mm. As a result, the die tip is very fragile and has to be replaced frequently once the metal between the orifices is broken
- The air manifold, shown in Figure 5, supplies hot, high-velocity air which draws the polymer filaments into much thinner microfibres. The manifolds are unusually located at the side of the die nosepiece, and the hot air comes in contact with the polymer as it exits the die tip. The air is hot than the polymer to ensure the polymer remains liquefied during the process

Both the spunbond and melt-blown technologies are capable of processing a great variety of thermoplastic like polypropylene, polyester, polyethylene, polyamide, and polyurethane [57, 58, 60]. Of all materials, polypropylene is the most common as it is relatively cheap and has low melt viscosity for easy processing. Coincidentally, polypropylene is the most common material used for a 3-ply surgical mask while other materials like polystyrene, polycarbonate, polyethylene, and polyester can also be used in masks [62].

### 3.2. Air Flow Through Mask

**Figure 6:**
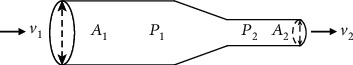
Air flows through a channel with different sizes.

To understand the flow of air through the mask, fluid mechanics is needed.  Figure 6 illustrates how air flows through a narrow channel.

According to the Bernoulli equation,

(1) $$\begin{array}{c} P_{1}+\frac{1}{2}\rho v_{1}^{2}+\rho gh_{1}=P_{2}+\frac{1}{2}\rho v_{2}^{2}+\rho gh_{2}, \end{array}$$

where *P* is the pressure, *ρ* is the density of the fluid, *v* is the velocity, *g* is the gravitational constant, and *h* is the height.

Under restrictions:

1. Steady flow
2. Incompressible flow
3. Frictionless flow
4. Flow along a streamline

Since the channel in Figure 4 is along the same horizon,

(2) $$\begin{array}{c} \;\rho gh_{1}=\;\;\rho gh_{2}. \end{array}$$

Combining (1) and (2),

(3) $$\begin{array}{c} P_{1}+\frac{1}{2}\rho v_{1}^{2}=P_{2}+\frac{1}{2}\rho v_{2}^{2},\\ P_{2}-P_{1}=\frac{1}{2}\rho v_{1}^{2}-\frac{1}{2}\rho v_{2}^{2},\\ \Delta P=\frac{1}{2}\rho \left(v_{1}^{2}-v_{2}^{2}\right),\\ \Delta P=\frac{1}{2}\rho \left(v_{1}+v_{2}\right)\left(v_{1}-v_{2}\right). \end{array}$$

Under the conservation of mass, the continuity equation ensures that the amount of mass entering the channel is equal to the amount of mass exiting the channel:

(4) $$\begin{array}{c} {\rho A}_{1}v_{1}={\rho A}_{2}v_{2},\\ \frac{A_{1}}{A_{2}}=\frac{v_{2}}{v_{1}}, \end{array}$$

where *A* is the area and *v* is the velocity.

Therefore, when air flows through the channel in Figure 6,

(5) $$\begin{array}{c} \frac{A_{1}}{A_{2}}=\frac{v_{2}}{v_{1}}>1. \end{array}$$

When Equation (5) > 1, the change in pressure, Equation (3), when the air flows through the channel in Figure 6 is

(6) $$\begin{array}{c} \Delta P<0. \end{array}$$

**Figure 7:**
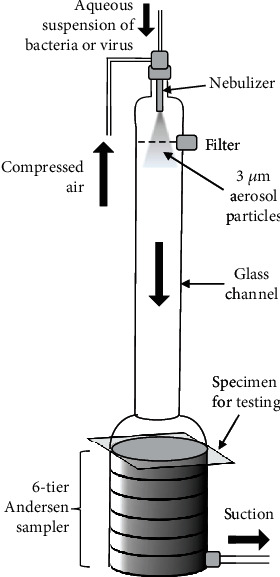
Design of the setup for evaluating BFE and VFE of mask materials using a six-stage Andersen sampler.

The airflow through the channel in Figure 7 illustrates how air will penetrate the mask whenever the user breathes in. Air from the surrounding environment is forced into the numerous tiny pores on the mask. From Equations (3) and (5), it is clear that the larger the difference in the areas the air flows through or the smaller is the mask pore, the greater the pressure drops or negative differential pressure. In other words, the more difficult it is for the wearer to breathe through the mask.

### 3.3. Performance Criteria for Commercial Masks

Face masks provide the user with protection against airborne particles, pathogens, secretions, and body fluids by physically filtering them from breathable air. According to the American Society of Testing and Materials (ASTM) F2100 standard, which specifies the performance requirements for materials used in medical face masks [63], five performance characteristics have been identified. These are particulate filtration efficiency (PFE), bacterial filtration efficiency (BFE), fluid resistance, differential pressure, and flammability. As face masks are an integral part of the personal protective equipment (PPE) kit for medical use, these standardized characteristics ensure consistency in mask production and testing validation and help the end-user to make the most informed choice of mask for the intended application.

#### 3.3.1. Particulate Filtration Efficiency (PFE)

This test measures the filtration efficiency of face masks towards monodisperse particles under a constant airflow rate. For PFE testing, 0.1 *μ*m polystyrene latex particles are used according to FDA guidance [64] at airflow velocities of 0.5-25 cm/s as recommended by the ASTM F2299 standard, for quantifying the filtration efficiency of materials used in facial masks [65]. Light scattering is used to quantify the particle count in the upstream feed (*M*_u_) prior to filtration, as well as that in the downstream filtrate (*M*_d_). The filtration efficiency (*E*), often expressed as a percentage, can be calculated with Equation (1):

(7) $$\begin{array}{c} E=100\left(1-\frac{M_{\text{d}}}{M_{\text{u}}}\right). \end{array}$$

The percentage of penetration (*P*), or leakage of the particles through the mask medium, can hence be quantified by

(8) $$\begin{array}{c} P=100\left(\frac{M_{\text{d}}}{M_{\text{u}}}\right)=100–E. \end{array}$$

It thus follows that the higher the value of *E*, with a corresponding smaller *P*, indicates a better ability of the mask material to filter submicron particles. While the F2299 standard allows consistent comparison of the PFE value of different materials used for face masks, it does not access the effectiveness of the overall design of the face mask, nor the quality of the mask's seal to the wearer's face.

#### 3.3.2. Bacteria Filtration Efficiency (BFE)

This test quantifies the performance of the mask material in filtering out bacteria when challenged with an aerosol of *Staphylococcus aureus*, as recommended by the ASTM F2101 standard [66]. *S*. *aureus* was chosen for its clinical relevance as one of the leading causes of nosocomial infections acquired in a hospital or healthcare facility [67, 68]. To perform the test, an aerosolized liquid suspension of *S. aureus* (mean particle size of 3.0 ± 0.3 *μ*m) is delivered to the target filter sample at a constant flow rate of 1 ft^3^/min (or 28.3 L/min). As shown in Figure 7, the aerosol is then drawn through a six-stage Andersen sampler [69]. Each tier contains an agar plate which acts as a medium for the growth of any bacteria which passes through the filter material to form visible colonies on the plates. A control is also performed under identical conditions in the absence of the filter specimen. The percentage BFE can be calculated by the formula:

(9) $$\begin{array}{c} \text{BFE}=100\left(\frac{C-F}{C}\right), \end{array}$$

where *C* and *F* represent the number of bacteria colonies in the control and in the presence of the filter, respectively. Using the ASTM F2101 standard, a maximum BFE of 99.9 % can be achieved.

For surgical masks, a minimum BFE of 95% BFE is required. It should be noted that other than the ASTM specifications, some mask manufacturers quantify BFE ratings with the modified Greene and Vesley method [70], which measures the effectiveness of the mask in preventing bacteria from passing through when worn on a human test subject's face. This method is not comparable with ASTM F2101 and is not recommended by ASTM for comparison [71]. The ASTM F2101 method possesses numerous advantages, including a highly reproducible testing procedure, the ability to tightly control the mean bacteria aerosol particle size, and has not been modified for many years, which provides a consistent set of standards for comparing across many different filter materials assessed at different times [72]. However, like the ASTM F2299 standard for PFE, the ASTM F2101 standard for BFE does not evaluate the fit, design, and facial-sealing properties of the mask.

#### 3.3.3. Viral Filtration Efficiency (VFE)

The viral filtration efficiency (VFE) is another parameter used by mask manufacturers for marketing and in FDA 510(k) applications for certain N95 filtering facepiece respirators [73], although it is not currently recognized as a standard test method by ASTM and hence is not a requirement for mask evaluation. The VFE test utilizes the same procedure and setup as recommended by ASTM F2101 for BFE (Figure 7) [72]. The bacteriophage *Φ*X174, which infects only *E. coli* bacteria, is used as the challenge virus that is aerosolized to form 3.0 ± 0.3 *μ*m virus-containing water droplets (not individual viruses). Unlike the BFE test, the agar plates in the Andersen sampler are first inoculated with *E. coli*, and areas in contact with the viral droplets become clear as the bacteria cells are lysed to form plaques. The VFE value is calculated by comparison with a control without the filter material as described above for BFE.

#### 3.3.4. Fluid Resistance

Fluid resistance evaluates the mask's ability to act as a barrier to the transfer of fluids from its outer to its inner layers due to spraying or splashing. According to the ASTM F1862 standard, 2 mL of synthetic blood, containing a red dye for visual detection and a thickening agent for stimulating blood flow properties, is dispensed against a complete medical mask specimen at different velocities [74]. These velocities correspond to different blood pressures of 80 mmHg (Level 1, venous blood pressure), 120 mmHg (Level 2, arterial pressure), and 160 mmHg (Level 3, high pressures occurring during trauma or under surgical conditions with high-pressure irrigation) [71], assuming the face mask is within 300 mm of the blood vessel puncture. The pass/fail determinations are based on visually detecting penetration of the synthetic blood to the inner layer. To simulate actual usage conditions, i.e., breathing, which creates high humidity (thus affecting fluid resistance), and mask material, the test specimens are also preconditioned at high relative humidity of (85 ± 5)% at (21 ± 5)°C.

#### 3.3.5. Differential Pressure (DP)

This parameter, otherwise known as “delta P,” measures the ability of the mask material to restrict airflow through it, giving an objective indication of the mask's breathability. Typically, it is determined by measuring the difference in air pressure on both sides of the mask material using a manometer at a constant airflow rate, and the difference in pressure is divided by the surface area of the sample, according to the MIL-M-36954 standard [75]. As such, DP is usually expressed in units of mm H_2_O/cm^2^, where a lower value (i.e., smaller difference in pressure on both sides) indicates greater breathability, feels cooler to the wearer, and hence gives an overall better comfort level. ASTM requires that moderate and high barrier masks have a DP value of <5.0, while low barrier masks have DP < 4.0. It is noteworthy that a trade-off exists between DP and fluid resistance for the same design and fit of the wearer: generally, an increase in resistance to synthetic blood penetration also results in a greater pressure drop across the mask layers and hence reduces breathability [74].

#### 3.3.6. Flammability

Hospitals contain numerous sources of ignition, such as heat, oxygen, and fuel sources. As the natural and synthetic fibres making up the mask materials are flammable, these can pose potential risks to the wearer due to the speed and intensity of flame spreading. Mask flammability is assessed in accordance with the 16 CFR Part 1610 standard, typically performing the tests on 5-10 test samples [76]. In a nutshell, the mask specimen is first cut into the defined dimension of 50 × 150 mm, then mounted and secured onto a specimen holder. Thereafter, the mounted specimen is conditioned in a desiccating oven at (105 ± 3)°C for 30 minutes, before it is then transferred to the test chamber. A stable butane flame of fixed length (16 mm) is then impinged upon the sample for exactly 1.0 s. The burn time, i.e., the time taken for the flame to travel up the specimen till a stop device is triggered, is then registered. According to the ASTM F2100 Standard for Performance of Materials Used in Medical Face Masks [63], the masks need to meet the requirements of Class 1 flammability, with an average burn time of ≥3.5 s [77].

In addition to these aforementioned standardized tests, the medical face masks should be tested according to ISO 10993-5 and 10, which specifies cytotoxicity [78] and skin sensitivity [79] test methods, respectively, to ensure the materials are not harmful to the wearer. A summary of various mask types, their performance criteria, and use applications is provided in Table 1.

## 4. Masks Made from Household Materials

The surge in demand worldwide for commercial face masks during the COVID-19 pandemic has led to a global shortage of supplies for both physical products as well as raw materials [80]. In this circumstance, making a mask at home can be a life-guarding action [81]. Homemade masks may vary from the commercial ones in terms of structural integrity and effectiveness, but they are cheap and accessible. Wearing a simple cloth mask is far better than wearing no mask to safeguard the wearer and the others' health [81, 82].

Using commonly available household materials, it is easy to fabricate simple masks that may block respiratory droplets from the wearer. A lot of household materials have been used to fabricate masks and tested accordingly. These typically include cotton fabrics, clothing, silk, tissue paper, kitchen towels, pillowcase, and tea cloths. In the H1N1 influenza pandemic, researchers tested the efficiency of homemade masks against that of commercial masks. van der Sande et al. designed a series of experiments, including short-term (10–15 mins) inward protection, long-term (3 hrs) inward protection, and outward transmission prevention, to compare the effectiveness of three types of masks under different movement activities [14]. An N95-equivalent Filtering Facepiece against Particles- (FFP-) 2 mask (1872V®, 3M), a surgical mask (1818 Tir-On®, 3M), and a homemade mask made of TD Cerise Multi® tea cloths (Blokker) were chosen. In the short-term protection test, all masks gave some protection to both adults and children against airborne particles. FFP-2 provided the best protection to adults (25 times as much as a surgical mask and 50 times as much as a homemade mask from tea cloths), while the homemade mask provided the least protection. The protection to children was less efficient with all masks, though the efficiency ranks were the same as in adults. Activity (nodding, shaking, reading, and walking) had no obvious impact on efficiency. In the long-term protection test, the conferred protection remained highest with the FFP-2 mask and lowest with the homemade mask. Interestingly, the measured median protection factors increased with the wearing time for the homemade mask, while they decreased with the FFP-2. In the outward protection test, the mask type significantly determined the protection factors. The homemade mask only provided marginal outward protection, while the FFP-2 and the surgical mask, which performed similarly, provided better outward protection. Despite the relatively low effectiveness, it was suggested that wearing a homemade mask might sufficiently reduce viral exposure [12]. The marginal respiratory protection was also observed with masks made with other common materials including sweatshirts, T-shirts, towels, and scarves, when tested against polydispersed and monodispersed aerosols (20–1000 nm) [83]. Compared with the control N95 respirator, these fabric materials allowed higher penetration by aerosols, indicating poorer protection for wearers.

In addition to material, other factors, including the design, the velocity, the fitness to the wearer's face (sealing issue), and the properties of the particles to which it will be exposed, also affect the overall performance of a homemade mask. A more comprehensive study was conducted by Davies et al., to test the efficacy of homemade masks against bacterial and viral aerosols (Bacillus atrophaeus (*B. atrophaeus*) with a size of 0.95–1.25 *μ*m, and bacteriophage MS2 with a size of 0.023 *μ*m) [12]. The masks were made from different common household materials, including 100% cotton T-shirt, scarf, tea towel, pillowcase, antimicrobial pillowcase, vacuum cleaner bag, cotton mix, linen, and silk. As shown in Table 2, all materials are capable of blocking the microorganisms to different degrees, and they all worked better in the case of *B. atrophaeus* due to its large size. Although the surgical mask as a control sample possesses the highest efficacy, the vacuum cleaner bag, tea towel, and cotton mix also showed filtration efficiency of higher than 70%. The ones with the lowest efficiency were the scarf, pillowcase, and silk, most of which however still had >50% efficacy. Another important factor that needs to be considered when using a face mask is the ease of breathing, which is indicated by pressure drop. The higher the pressure drop, the higher the difficulty for the wearer to breathe. It is obvious that despite the high filtration efficiency of a vacuum cleaner bag and tea towel, their high-pressure drop values make them unsuitable for masks. Combining the above two factors, it was suggested that the most suitable household materials for a homemade mask are pillowcase and 100% cotton t-shirt, and further studies showed that doubling the layer did not help improve the efficacy significantly [12]. Yet, doubling increased the pressure drop, indicating more difficulty for breathing. This work again provides the insight that homemade masks are capable of blocking bacteria and viruses to some extent, yet their overall performance (filtration efficiency, pressure drop, and fitness) is not comparable to N95 and surgical masks. Indeed, wearing a mask can reduce the infection probability yet cannot eliminate the disease. It must be implemented community-wide [18] and together with multiple nonpharmaceutical preventative measures, such as hand hygiene, social distancing, quarantine, and immunization, to minimize the transmission and stop the outbreak [84]. In that sense, the homemade masks may be the last resort when facing a supply shortage, and they may well protect the general public.

Historically, cloth masks have been used to protect healthcare workers (HCWs) from respiratory infections [85–87], yet it is only in recent years that researchers started to systematically study their efficacy. Chughtai et al. reviewed the use of cloth masks [88] and conducted a series of studies including randomized clinical trial (RCT) to evaluate how good the cloth masks are to protect HCWs [47, 89]. Finding that the rate of respiratory infection was highest in the cloth mask group and that the particle penetration of cloth masks was 97% (versus 44% for medical masks), the authors concluded that cloth masks should not be recommended for HCWs, especially in highly infectious situations. Shakya et al. examined the efficiency of a cloth mask against monodispersed polystyrene latex (PSL) particles (30 nm to 2.5 *μ*m) and diluted whole diesel exhaust [90]. It was found that with an exhaust valve, the cloth mask had a filtration efficiency of 80-90% against PSL particles. Without a valve, the efficiency against the same PSL particles drops to 36-65%, although the cloth mask performed better against larger particles. The cloth mask's filtration efficiency ranged from 15 to 75% against whole diesel particles. The overall performance results suggested that cloth masks provided marginal protection to the wearer from particles less than 2.5 *μ*m. Furthering the study on filtration efficiency, Neupane et al. investigated the effect of washing and drying of cloth masks on the filtration performance and correlated the performance to the pore size and shape in the masks [91]. It was found that the PM_10_ filtration efficiency dropped by 20% after the 4^th^ washing and drying cycle, which was ascribed to the increase in pore size and the lack of microfibres within the pore region. Long-term usage of the cloth masks entails continuous stretching of the mask, enlarging the pore size, thus impairing mask performance.

**Figure 8:**
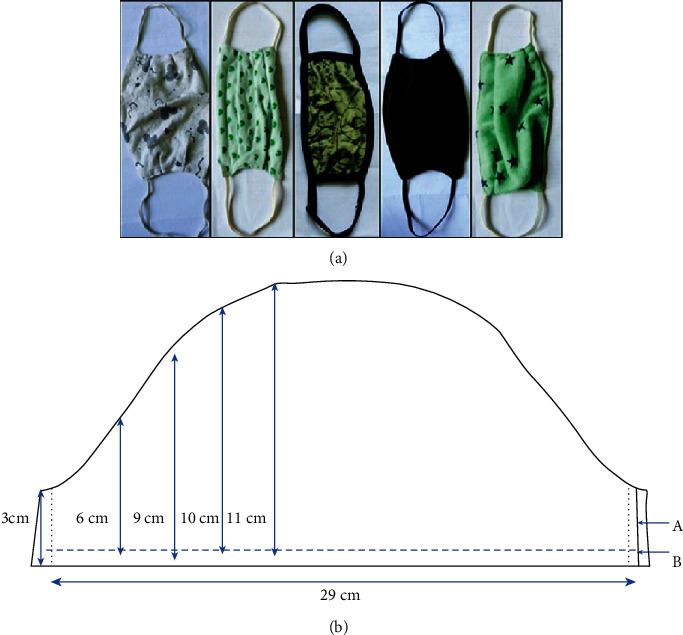
(a) Photos showing examples of simple cloth masks. Reproduced with permission from Ref. [91]. Copyright 2019, Neupane et al., PeerJ. (b) Schematic showing the pattern for a homemade mask. Reproduced with permission from Ref. [92]. Copyright 2020, Springer Nature.

**Figure 9:**
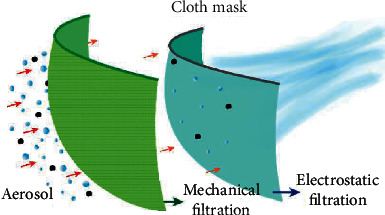
A new design of homemade cloth masks from common fabric materials. Reproduced with permission from Ref. [95]. Copyright 2020, the American Chemical Society.

Making a cloth mask can be as simple as combining two cloth layers with stretchable ear loops (Figure 8(a)) [91]. Sugrue et al. introduced a step-by-step method of making a cloth mask using household materials including cotton, metal garden wire, and elastic bands (Figure 8(b)) [92]. The fabricated cloth mask has demonstrated its good comfort and fitness to the human face. While one can also find instructions on how to sew a fabric face mask at home [93, 94], Konda et al. recently developed a new model to fabricate homemade cloth masks that can achieve high filtration efficiency against aerosol particles ranging from 10 nm to 10 *μ*m in size [95]. By combining different commonly available fabrics, for instance, cotton-silk, cotton-chiffon, cotton-flannel, and filtration efficiency for particles < 300 nm and >300 nm can be as high as >80% and >90 %, respectively. The high efficiency comes from the synergistic effect of mechanical filtration from cotton and electrostatic filtration from the other layer like silk (Figure 9). It was also highlighted in this work that for the same material such as cotton, there are other factors that critically and significantly affect the overall performance when used as a mask. These include the layer number, the layer density (threads per inch, TPI), and the facial fitness (openings and gaps between the mask edge and the facial contours). Therefore, future mask development should consider the above factors while taking into consideration the breathability, washability, and reusability.

## 5. Decontamination of Face Masks

The worsening and prolonging of the COVID-19 pandemic have led to a surge in daily consumption and demand of PPE items, including face masks, by frontline healthcare workers, which resulted in a global shortage of face masks and raw materials. Unfortunately, the production of masks cannot be easily ramped up to meet this sudden surge in demand. While reusable cloth masks purchased or self-made from household materials can serve as a substitute for disposable surgical masks among the general population, such masks do not offer sufficient protection against the virus for healthcare workers who are working and in constant contact with infected patients for prolonged hours. In fact, these workers require the wearing of filtering facepiece respirators (FFR) such as the N95 masks as part of their PPE, which offer even more protection against airborne pathogens compared to the normal disposable 3-ply surgical masks. These masks are meant to be of one-time use, i.e., disposable, and are not recommended to be reused. The shortage of supply faced by many countries and hospitals, particularly those without sufficient stockpile, has however resulted in desperate measures taken, including the decontamination and reuse of face masks.

Research efforts have been invested into finding the best possible way of decontaminating used FFRs for reuse purposes. Several decontamination methods have been shortlisted and even implemented in some hospitals, including the use of ultraviolet (UV) radiation, hydrogen peroxide, ethylene oxide, steam, and heat, which will be discussed in greater details herein. The requirements for an effective decontamination method for masks and FFRs are as follows: (i) pathogens contaminated on the surfaces of masks and FFRs must be effectively killed and inactivated; (ii) there must no reduction in the filtering performance of masks and FFRs towards pathogens and particulates; (iii) structural integrity of all other components of the masks and FFRs (including elastic straps and metallic noseband) must not be adversely affected; (iv) for FFRs, tight-fitting to the users' face must not be compromised; and (v) decontamination must not leave behind chemicals or by-products that may affect the health and well-being of users. In addition, other considerations have to be taken to ensure the decontamination method can be practically carried out in a sufficient scale, by hospitals or individuals at home. These include (i) the availability and cost of resources, including space, equipment, and chemicals; (ii) the ease and robustness of the process; (iii) safety to the person performing the decontamination process, especially when it dues to with harmful radiation or chemical fumes; and (iv) the scale at which the process can be performed. Research efforts in developing and optimising mask decontamination methods therefore commonly involve (i) testing the effectiveness of the methods in killing different pathogens coated over mask surfaces; (ii) testing the filtering performance, fit factor, and structural integrity of masks after decontamination; and (iii) determining how many cycles of decontamination processes can the masks undergo before deteriorations were detected.

Several review articles and websites have effectively summarised research findings and outcomes derived from efforts in this area [96–98]. For instance, the webpage N96DECON provides online resources on methods of decontaminating N95 masks [99], whereas the CDC of the USA has dedicated a webpage making recommendations on appropriate FFR decontamination methods [3]. In addition, recent research efforts have also been undertaken to test these methods for their effectiveness against the SARS-CoV-2 virus, given many of these methods were reported before the COVID-19 pandemic and tested against a range of different bacteria and viruses. In general, there are many ways and chemical agents (e.g., bleach and soap) that can be used to disinfect pathogens on our hands, small items, and common surfaces of high touchpoint, but not all of these can be practical for mask decontamination.

### 5.1. Decontamination by UV Radiation

Short wavelength UV radiation (UV-C, *λ* = 254 nm) is commonly used to disinfect small items, which can kill pathogens in a matter of minutes of exposure. UV radiation has been commonly adopted to disinfect medical items in hospitals, vehicles in bus and train depots, scissors and combs in barbershops, and even baby milk bottles at home. Ultraviolet germicidal irradiation (UVGI) has therefore been one of the most commonly studied and adopted methods for the decontamination of masks and FFRs. The effectiveness of UVGI in mask decontamination depends on three important factors: (i) the intensity of UV radiation, (ii) the duration of exposure, and (iii) the dimension and direction of UV radiation with respect to the mask. For the first two factors, prolonged exposure to very high-intensity UV radiation may result in the degradation of mask materials, which may compromise the filtering capacity and the mask-fitting (for FFRs). An optimal radiation intensity and treatment duration must therefore be fine-tuned. For the third factor, the even exposure of all possible surfaces of the mask to UV radiation will be ideal for thorough decontamination, but this may be challenging based on how users position both the UV sources and the masks in the disinfection chamber.

To study the effects of UVGI treatments on N95 respirators, Viscusi et al. exposed FFRs, 15 minutes on each side, to 176–181 mJ/cm^2^ of UV radiation [100, 101]. No visible changes were observed to the FFRs, while both the filter aerosol penetration and filter airflow resistance were not affected as well. Bergman et al. evaluated the effects of three 15-minute UVGI treatment cycles (1.8 mW/cm^2^) on FFRs [102, 103]. No degrading effects on filtration performance or face-fitting were observed for the different models of FFRs tested as all treated masks managed to pass quality and safety standards. Lindsley et al. reported that there was a small increase in particle filtration performance (up to 1.25%) but little effect on flow resistance for most models of FFRs upon undergoing UVGI treatment with an exposure dose between 120 and 950 J/m^2^ [104]. The elastic strap, however, experienced decreases in breaking strength as the dosage of UV radiation increases. More recently, Liao et al. reported that face masks can maintain filtration efficiency above 95% after 10 cycles of UVGI treatment (∼3.6 J/cm^2^), only to show small degradation to ~93% after 20 cycles [105].

The decontamination effectiveness of UVGI treatment was studied. Earlier, Fisher and Shaffer investigated the decontamination effects of UV treatment on the different layers of masks by exposing different models of N95 FFRs exposed to MS2 coliphage to UV-C radiation at a minimum dose of 1000 J/m^2^ for durations of 2 to 266 minutes [106]. The porous nature of N95 FFRs' materials allows UV radiation to penetrate through different layers, thus registering at least a 3-log reduction of viable MS2. Heimbuch et al. demonstrated the effectiveness of UVGI treatment (15-minute exposure at a dose of 18 kJ/m^2^) on N95 FFRs contaminated with H1N1 aerosols and droplets, where an average log-reduction of 4.69 and 4.92 was recorded, respectively [107]. In a separate study, Mills et al. reported more than a 3-log reduction in H1N1 virus viability in 12 and 7 facepieces and elastic straps, respectively, out of 15 UVGI-treated (1-minute exposure at a dose of 1 J/cm^2^) N95 FFRs of different models [108]. More recently, Fischer et al. and Ou et al. assessed the feasibility of UVGI for decontaminating SARS-CoV-2-contaminated masks. The former concluded that UV-irradiation at wavelength 260–285 nm can effectively sterilize the N95 FFRs up to three cycles with no compromise to mask performance [109], whereas the latter reported the that N95 FFRs and surgical masks may be effectively decontaminated at an exposure dose of 216 mJ/cm^2^ for 5 minutes for up to 10 cycles, without significant deterioration of filtration efficiency and fit factor [110].

While UV radiation is harmful to human skin and eyes, UVGI treatment of contaminated masks serves as an attractive decontamination method which avoids the use of toxic chemicals. In a hospital in Nebraska (USA), for example, decontamination was performed by having contaminated masks hanged on wires drawn across an empty room fitted with two UV light towers [111]. A total of 2000 masks can be sterilized a day based on this UVGI treatment method. Nonetheless, one drawback to UVGI treatment is the unlikeliness in achieving homogenous decontamination across the entire exterior surface of the masks and FFRs due to the UV light not being able to reach the “shadowed areas” and crevices attributed to the positioning of masks with respect to the UV source, and multiple pieces of a mask being treated together [96].

### 5.2. Decontamination by Hydrogen Peroxide Vapour

Hydrogen peroxide (H_2_O_2_) solution is a common antiseptic in wound treatment whereas H_2_O_2_ vapour (HPV) is often generated to disinfect enclosed spaces, such as offices, workstations, hospital wards, and interiors of buses, trains, and aircraft, as well as to sterilize laboratory and medical equipment and tools in specially designed enclosed chambers. The generation of HPV by hospitals and cleaning companies for the disinfection of common spaces can be achieved with the use of portable “vaporizer” machines. Likewise, the easy generation of HPV at low temperatures makes it a desirable method for disinfecting masks and FFRs. Unlike UVGI, HPV is able to reach all “shadowed areas” and crevices of the mask exteriors during treatment, thus ensuring decontamination to be more thorough. Likewise, the decontamination effectiveness of vaporized H_2_O_2_ (VHP) treatment and its effect on mask performance and structural integrity depend on exposure time, VHP concentration, and treatment regime, which often involves dehumidification, conditioning, dwell, gassing, and aeration. In addition, H_2_O_2_ readily decomposes into oxygen and water so exposure to residual H_2_O_2_ is not a big issue. The VHP method is used in some hospitals due to the availability of H_2_O_2_ vaporizer machines. For instance, Hospitals in Ohio (USA) under OhioHealth worked with research organization Battelle Memorial Institute to retrofit enclosed rooms in hospitals for VHP decontamination, which can treat thousands of masks in one go [111].

In general, the VHP method is able to effectively decontaminate masks from different bacteria and viruses without compromising mask performance. Early works by Viscusi et al. reported that N95 FFRs undergoing VHP treatment for up to 55 minutes and temperature up to 80°C only exhibited slight tarnishing of metallic nosebands without significant changes to filtering capacity [100, 101]. Bergman et al. further confirmed that N95 FFRs undergoing 3 cycles of VHP treatment with HPV concentration of 8 g/m^3^ for 125 minutes (each cycle) do not yield any degradation to filtering performance as well, with recorded mean filter penetration of below 4.01% [102]. The nondamaging nature of VHP treatment was validated by a laboratory testing conducted by Battelle and funded by FDA (in the USA); N95 FFRs were found to still meet filtering performance and fit requirement even after 50 cycles of VHP treatment using a Bioquell Clarus C HPV generator, although elastic straps started to deteriorate [112]. More recently, Schwartz et al. reported similar findings through the decontamination of 100 3M^TM^ N95 FFRs via treatment with 480 ppm of HPV, involving five stages: conditioning, pregassing, gassing (25 minutes), gassing dwell (20 minutes), and aeration, with no physical or performance degradation recorded [113].

In view of the COVID-19 pandemic, the suitability of VHP for the treatment of SARS-CoV-2-contaminated masks was studied. Kenney et al. demonstrated the effectiveness of VHP treatment on N95 FFRs, which demonstrated a complete eradication of 3 aerosolized phages (that mimicks the SARS-CoV-2): T1, T7, and *Pseudomonas* phage phi-6 in just one cycle of treatment, involving 10 minutes of conditioning, 30–40 minutes of gassing at 16 g/m^3^ of HPV, 25 minutes of dwelling, and 150 minutes of aeration [114]. No deformity was observed for the FFRs after 5 cycles of treatment. Similarly, Kumar et al. reported no recoverable SARS-CoV-2 viruses on N95 FFR surfaces after the masks undergo a one-hour treatment cycle involving 10 minutes of dehumidification, 3 minutes of conditioning, 30 minutes of decontamination, and 20 minutes of aeration. Throughout the process, the peak HPV concentration of 750 ppm and the FFRs can undergo 10 cycles of treatment without compromise to mask performance [115]. Likewise, Smith et al. also reported no functional degradation to N95 FFRs (both filtering capacity and fit factor) after two cycles of VHP treatments involving 20 minutes of gassing (~500 ppm), 60 minutes of dwelling (~420 ppm), and 210 minutes of aeration at ambient temperature, with no viable virus detected at the end of each cycle [116]. Finally, Fischer et al. concluded that among VHP, UVGI, heat, and ethanol treatment, VHP treatment displayed the best combination of rapid inactivation of SARS-CoV-2 and preservation of N95 FFR integrity [109].

### 5.3. Decontamination by Ethylene Oxide Vapours

There has been some early research work conducted on the viability of using ethylene oxide vapour to decontaminate FFRs. Viscusi et al. reported that treating FFRs with ethylene oxide vapour (725–883 mg/L) at 55°C for 1 hour, followed by 4 hours of aeration, do not yield any visible sign of mask degradation, with the exception of darkening of elastic straps [100, 101]. The treated masks passed filtration performance assessments. More recently, Kumar et al. reported that N95 FFRs undergoing 1 hr exposure to ethylene oxide vapour followed by 12 hours of aeration managed to achieve complete sterilization of SARS-CoV-2 viruses [115]. The masks can tolerate at least 3 cycles of treatment without any significant structural or functional deterioration. However, there were concerns about the toxicity of ethylene oxide towards mask wearers as it is potentially carcinogenic and teratogenic. In addition, ethylene oxide is flammable, which posed fire safety hazards to the treatment process as well. CDC does not recommend the decontamination of masks using this method [3].

### 5.4. Decontamination by Heat, Moist, and Steam

Heat, moist, and steam appear to be one of the most popular ways of decontamination because they do not require the use of dangerous chemicals and radiation or any sophisticated equipment. The simplicity of such treatments can even be replicated or performed at home with the use of microwave ovens, rice cookers, and steamers. For the use of microwave ovens, steam treatment may be carried out in a microwave steam bag, which is not an uncommon household item, given its uses in cooking or disinfecting items used for babies. The downside to this is method is that given the size of microwave ovens, the treatment method cannot be performed one mask at a time. There were also concerns that prolonged exposure to microwave radiation may cause deterioration of mask performance. On the other hand, people in Taiwan were advised to decontaminate their used surgical masks via “dry steaming” in a rice cooker for 3 minutes, which was said to achieve 99.7% sterilization rate although the mask's filter quality would deteriorate by 10% [117]. The effectiveness of decontamination via moist heat treatment varies according to temperature and relative humidity (RH). Exposing masks and FFRs to too high heat may compromise structural integrity and performance. There were also debates over the importance of moisture and humidity in the sterilization process. Li et al., for instance, demonstrated that *Staphylococcus aureus* and MS2 phage inoculated masks experienced <3 and >5 log-reductions in both pathogens upon undergoing 15 minutes of dry heating and steam treatment, respectively, thus reflecting the importance of humidity in decontamination [118].

Earlier, Viscusi et al. reported N95 FFRs that underwent dry heating in an oven at 80°C for 60 minutes do not yield any visible physical changes whereas heating at 160°C for 22 minutes caused the polypropylene masks to melt and become unusable [101]. On the other hand, partial melting and increased filter penetration were observed for FFRs that underwent 2 minutes of dry microwave treatment (750 W/ft^3^, 1 minute per side) [100]. Bergman investigated the effects of 3 cycles of mask decontamination treatments via microwave oven generated steam (MGS, 2 minutes each cycle) and moist heat incubation (MHI, 15-30 minutes each cycle, 60°C, 80% RH). MGS and MHI treatments were both found to cause partial separation of the inner foam nose cushion for some of the FFRs, whereas slight melting of the head straps was also observed for MGS-treated FFRs due to sparking in the microwave [102, 103]. More recently, Liao et al. concluded that decontaminating face masks by heating below 85°C under various humidity levels appears to be a promising, nondestructive method for the preservation of filtration properties in melt-blown fabrics as well as N95 FFRs [105]. Face masks treated at 85°C at 30% RH can undergo 50 treatment cycles without significant changes in the filtration efficiency. Recent studies from Ou et al. also concluded that filtration efficiency and fit factor of N95 FFRs were not significantly compromised after 10 repeated cycles of both 30-minute steam treatment as well as 30-minute oven-dry heating at 77°C [110].

Investigating the effectiveness of different sterilization methods, Heimbuch et al. reported that no H1N1 viruses survived warm moist heat (WMH) treatment (3 hr, 65°C, 85% RH), whereas sporadic viable viruses were detected after MGS (with a water reservoir, 2 minutes, 1250 W) due to homogenous delivery of warm moisture by the former versus nonuniversal distribution of steam by the latter [107]. Likewise, Fisher et al. reported more than 4 log-reduction in MS2 phage viability with just 45 seconds of MGS treatment [119]. Fisher et al. further evaluated the viability of using microwave steam bags to perform MGS decontamination of FFRs, where the method yielded 99.9% efficiency in killing MS2 phage with a filtration efficiency of posttreated FFRs remaining above 95% [120]. More recently, Ma et al. subjected face masks made of melt-blown polypropylene to steam treatments of different durations (20–120 minutes) and found that they were still able to effectively block 98 to 99% of aerosolized H120 virus [121]. Likewise, Xiang et al. reported that dry-heating contaminated surgical face masks and N95 FFRs at 60 and 70°C for an hour can effectively kill different bacteria and fungi pathogens, as well as inactivating H1N1 virus. In addition, no physical changes were observed for both surgical face masks and N95 FFR even after heating for 3 hours at 70°C, where their filtering capacity remains at 97 and 96%, respectively [122]. Similarly, Fischer et al. reported that SARS-CoV-2-contaminated N95 FFRs that underwent dry-heating at 70°C can achieve effective sterilization with negligible changes to mask fit factor, even after 2 cycles of treatment [109].

### 5.5. Decontamination by Disinfectant Solution Treatments

Treatment of contaminated face masks by soaking in disinfectant solutions may not be the most preferred way of mask decontamination. One reason is because it involves posttreatment drying of a mask which may not only take a long time but also cannot guarantee the complete removal of residual disinfectant chemicals, which may be unpleasant to smell and/or pose health hazards. In addition, many common disinfectant solutions may be detrimental to the mask's structural integrity, thus compromising its filtering performance and fit factor after treatment. Choosing the right disinfectant solution is therefore important to ensure that posttreated masks not only do not degrade in structure and performance but also ensure disinfectant can be thoroughly removed to avoid odour and health threats to mask users.

Early studies by Viscusi et al. and Bergman et al. suggest that treatments in liquid H_2_O_2_ were not found to affect the filtering performance of N95 FFRs [101, 102]. FFRs submerged in 3% H_2_O_2_ for 30 minutes were reported to not yield significant visual changes, but those submerged in 6% H_2_O_2_ experienced ink fading on the exterior [101]. Three cycles of treatments in 6% H_2_O_2_ were also reported to cause staples on the FFRs to oxidise to varying degrees [102]. The effectiveness of decontamination by liquid H_2_O_2_ was however not evaluated.

Bleach was found to be a less desirable decontaminating agent due to the issue of odour. Viscusi et al. reported an increase in average filter aerosol penetration in different models of FFRs soaked in 0.525, 5.25% bleach solutions, although penetration of N95 FFRs still remains within 5% threshold [100, 101]. Filter airflow resistance of N95 FFRs was not affected much as well after 30 minutes of soaking in 6% bleach [100]. Also, the strong smell of bleach can still be detected even after the overnight drying of the treated masks [100]. Similar findings were made by Bergman et al. for FFRs after undergoing 3 cycles of bleach treatment (30 min, 0.6%) [102].

Ethanol was reported unsuitable for use for mask decontamination. The soaking of contaminated masks in 70% ethanol was recently reported by Smith et al. to cause impairment in mask function within 30 minutes although no SARS-CoV-2 viral RNA could be cultured [116]. Similarly, rapid inactivation of SARS-CoV-2 by 70% ethanol treatment was also reported by Fischer et al. but a loss of structural integrity of the N95 FFRs was resulted [109].

Although handwashing with soap can effectively kill pathogens, treating contaminated FFRs with soap water may not be a practical idea. Viscusi et al. found that although no visible changes were observed for FFRs soaked in soap water for 2 and 20 minutes, average penetration was found to increase significantly [101]. This was attributed to the surfactant of soap removing charges on electret fibres of the filtering material that plays an important role in preventing aerosol and particulate penetration.

### 5.6. Decontamination by Other Methods

Other disinfection methods were studied for decontaminating used masks including the use of autoclaves and disinfectant wipes. Autoclave treatment is commonly used to sterilize medical tools and apparatus via pressurized saturated steam at 121°C. Viscusi et al. reported the increase in average penetration, as well as deformation, shrinking, stiffening, and mottling of N95 FFRs after 15 and 30 minutes of autoclave treatment [100, 101]. On the contrary, a recent study by Kumar et al. reported that masks with layered fabric, pleated models tolerated 10 cycles of autoclave treatment while maintaining structural and functional integrity, whereas masks with more rigid moulded models demonstrated loss of function after one autoclave cycle [115].

Disinfectant wipes are convenient to use but their effectiveness in decontaminating masks may not be ideal. Heimbuch et al. studied the effectiveness of 3 different wipes, containing benzalkonium chloride (BAC), 0.9% hypochlorite (OCL), and no active antimicrobial ingredients (inert), respectively, on mask decontamination [123]. After 30 seconds of wiping, it was found that BAC- and OCL-wipe can only achieve 3 to 5 log-reduction in pathogens whereas inert wipe can only achieve ~1 log-reduction. Although average penetration remains below 5% after the use of all three wipes, there was an issue of uneven surface cleaning across different components of the mask [123].

Surgical masks and FFRs are made to be used only once and not meant to be reused. Nonetheless, should the need of reuse arise due to the issue of shortage, several well-studied decontamination methods can be considered. Among them, UVGI, VHP, and moist heat treatment appear to be more effective and suitable for large scale decontamination of mask in the hospital setting, whereas moist heat, dry heat, and steam treatment can be performed easily at home. Some other methods may not be practical as they may result in the compromise of structural integrity and degradation of performance. In fact, mask producer 3M has issued online technical bulletins to advise on the suitability of different models of FFRs for different methods of decontamination treatments [124]. Even more important, there is a need for proper handling of contaminated masks in the process of preparing for decontamination. Cautions must be practiced to avoid physical contact with a contaminated surface or exposure to viral-loaded aerosols that may be generated in the process. It is therefore advisable to wear protective gloves, masks, and even goggles when performing mask decontamination and to wash hands thoroughly with soap or disinfectant after that.

## 6. Engineering of Multifunctional Masks and Mask Materials

While existing models of masks and respirators serve users well in terms of the level of protection they offer against airborne pathogens, there has still been intensive research and developmental efforts to improve their filtering properties and performances, as well as comfort and user-friendliness. Such efforts can be categorised into two aspects: (i) improving the filtering capacity of mask material and (ii) engineering additional functions and properties into the designs of masks. The former involves material development and engineering—how do we process bulk materials to reduce their pore sizes such that they are sufficiently small to capture and filter off minute particulates and pathogens, and how do we treat or develop these materials to enable them to inactivate microorganisms. The latter involves making changes to the existing design of mask models—to confer on their antimicrobial properties through the application of coatings, for instance, and improve user comfort, friendliness, and convenience such as the introduction of sensing and self-cleaning properties.

### 6.1. Improving Air Filter Performance for Particulate Matter Capture

High-performance air filters should be able to capture particulate matter (PM) tiny particles with high efficiency while maintaining air permeability. Currently, the existing commercial air filters for PM capture are mainly composed of a mat of randomly arranged polymer fibres or fibreglass with diameters ranging from several microns to tens of microns. These filters always function by passively trapping PM which highly depends on the porous structure of the fibrous membrane. In order to achieve high removal efficiency, thick layers of densely packed fibres are needed and air permeability is therefore sacrificed. Recently, a variety of novel membrane filters have been developed for PM purification with enhanced performance and attractive characteristics such as smaller fibre diameters, higher specific surface area, low air resistance, and transparency, while being light and having functionalized active surfaces to capture particles more efficiently by electrostatic forces or chemical bond interactions. In this section, we will introduce the most popular innovative air filtration materials developed in the past five years for PM capture, including polymer nanofibre membranes, electret membranes, and porous metal-organic framework- (MOF-) based filters.

#### 6.1.1. Polymer Nanofibrous Membranes

The key factors affecting the functions of air filters are fibre diameter, membrane thickness, and air permeability. When the fibre diameter is decreased to nanoscale, the PM removal efficiency can be greatly improved due to enhanced specific surface area and high porosity, and thus, the membrane thickness can be reduced to ensure low air resistance. Among all the methods for fabricating nanofibrous membranes, electrospinning is the most widely used versatile technique which produces continuous nanofibres through an electrically charged jet of polymer solution [125]. Owing to the large specific surface area and highly interconnected porous network, electrospun membranes have been extensively employed for water treatment, biomedical and energy-related applications over the past decades, and they have recently attracted renewed research interest since Lui's group demonstrated the high efficiency of electrospun fibres for air purification [126]. A transparent polyacrylonitrile (PAN) nanofibre membrane with an average fibre diameter of ~200 nm was fabricated by electrospinning and evaluated for the capture of PM_2.5_ particles. Compared with existing commercial air filters made of thick layers of micron-sized fibres which balance air resistance and filtering performance, the obtained nanofibre membranes displayed good optical transparency (up to 90%), high filtration efficiency (>95%), low-pressure drop (down to 132 Pa), and light weight. Strong adhesion of PM to the PAN nanofibre surface was demonstrated by the observation of *in situ* PM capture process. The developed transparent thin filter can be applied to indoor air protection through windows or incorporated into existing personal masks. Inspired by Lui's promising results, a variety of electrospun nanofibre membranes with different surface chemistry and mechanical or thermal properties have been developed from polymers, polymer blends, or polymer composites with surface-functionalized inorganic nanofillers for air purification investigations, including polyurethane [127], polycarbonate [128], poly(vinyl alcohol) [129], polytetrafluoroethylene [130], polybenzimidazole [131], polyacrylonitrile/polysulfone [132], polypropylene/polyethylene [133], polyurethane/polysulfonamide [134], polyacrylonitrile/graphene oxide [135], and polyacrylonitrile/MXene [136]. Besides conventional electrospinning, polymer nanofibre membranes mass-produced by needless electrospinning [137] and solution blow spinning [138] also demonstrated effectiveness for the capture of particulate pollutants.

**Figure 10:**
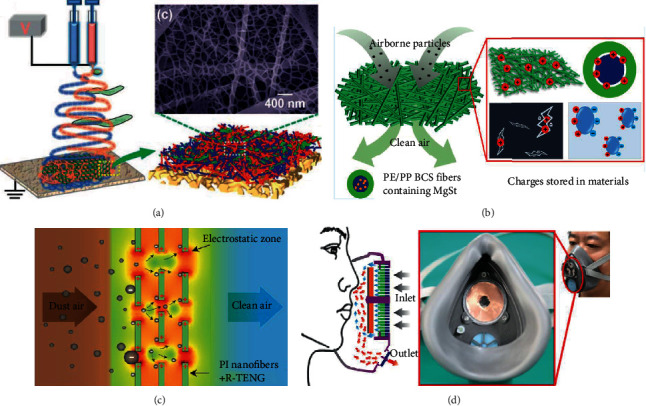
(a) Schematic showing the fabrication of nylon-6/PAN nanofibre/net membranes by electrospinning/netting technology. (b) Schematic showing an electret filter consists of PE/PP nanofibre as the matrix and magnesium stearate as the charge enhancer. (c) Schematic image of the filtration mechanism of the filter with R-TENG. (d) Structure and photo of a self-powered electrostatic adsorption face mask with R-TENG. Reprinted with permission from Ref. [126], copyright 2015, Macmillan Publishers Limited; Ref. [139], copyright 2015, The Royal Society of Chemistry; and Ref. [140–142], copyright 2017–2019, American Chemical Society.

To further improve capture efficiency towards ultrafine particles and reduce weight and packing density of the filters to ensure low resistance to airflow, polymer membranes with a novel nanofibre/net hierarchical porous structure have recently been developed using the cutting-edge electrospinning/netting technology, which is a versatile one-step process for fabrication of polymer membranes comprising common electrospun nanofibres interconnected with two-dimensional nanonets [143]. The nanonets, which are formed from the small charged droplets other than electrospinning jets under high electric field, exhibit amazing characteristics such as ultrafine diameter (<20 nm), high porosity, small pore sizes (<200 nm), and large specific surface area, making them attractive candidates for fine particulate filtration. In 2015, Wang's group fabricated ultralight nanofibre-nets binary nylon 6-PAN with high coverage (>98%) of nylon nanonets and low packing density of PAN nanofibres (Figure 10(a)) [139]. The prepared interconnected membranes displayed high filtration efficiency (99.99%) towards 300 nm aerosol particles with a low basis weight of 2.94 g m^−2^ and satisfactory quality factor (0.1163 Pa^−1^) under a high flow rate (90 L min^−1^), which is significantly superior to that of commercial glass fibre and melt-blown polypropylene fibre-based filtration membranes. Later, the same group further developed a highly integrated multilayer air filter comprising polysulfone microfibre (diameter ∼1 *μ*m), polyacrylonitrile nanofibre (diameter ∼200 nm), and polyamide-6 nanonets (diameter ∼20 nm) via sequential electrospinning [144]. The integrated filter with gradually varied pore structures and high porosity can efficiently capture airborne particles in a gradient manner with low air resistance. Very recently, Li et al. also employed the electrospinning/netting process for the mass production of a reusable poly(vinylidene fluoride) nanofibre/nanonet air filter, which presented a high purification efficiency of 99.985% towards PM_0.26_ and a low-pressure drop of 66.7 Pa [145].

#### 6.1.2. Electret Membranes

Unlike the passive membranes that capture particles mainly by the porous structure, the electrostatic air filters are able to effectively trap particles in an active manner with a larger attraction distance. Without depending on the high density of small pores, the filter thickness can be reduced and the removal efficiency can be maintained under a continuous airflow with a low-pressure drop. Three charging techniques, namely, *in situ* charging, corona charging, and tribocharging, can be used to fabricate electret membranes [146].

The electrospinning process for the fabrication of nanofibrous membranes can *in situ* charge the nanofibres through introducing charge storage enhancers into electrospinning solutions. Nanoparticles, such as polytetrafluoroethylene, silicon nitride, magnesium stearate, titanium dioxide, boehmite, and SiO_2_, are usually employed as charge enhancers, and various hybrid electret filters have been developed via the *in situ* charging technology of electrospinning [140, 147–149]. An electrospun polyethylene/polypropylene bicomponent membrane containing magnesium stearate (Figure 10(b)) was endowed with the elevated surface potential of 4.78 kV and exhibited a high filtration efficiency of 98.94% towards PM_2.5_ with a low-pressure drop of 37.92 Pa and excellent dust holding capacity of 10.87 g m^−2^ [140]. A polyvinylidene fluoride nanofibrous membrane doped with well-dispersed SiO_2_ nanoparticles demonstrated a remarkable electret effect with a surface potential of 12.4 kV and high filtration performance towards particles with different sizes [149]. Besides electrospinning, corona treatment is another approach to charge fibrous membranes under an external electric field. Zhang et al. fabricated electret polypropylene nonwovens via melt blowing followed by corona charging with magnesium stearate as the charge enhancer [150]. After being charged at a voltage of 100 kV for 30 s, the electrostatic nonwoven filter demonstrated high filtration efficiency up to 99.22% against PM_2.5_, low-pressure drop of 92 Pa, and satisfactory QF value of 0.054 Pa^−1^.

A fatal drawback of the *in situ* and corona-charged electret membranes is the poor stability of filtration efficiency due to the rapid dissipation of the surface charges when the membranes are in contact with moisture or oil droplets under a hazy environment [151]. Electret membranes fabricated by a tribocharging strategy with constant charge supply can solve the problem of charge dissipating, leading to enhanced stability and prolonged service life of the filters. Very recently, a triboelectric nanogenerator (TENG) has been combined with nanofibrous air filters for high-efficiency particulate removal [141, 142]. TENG is a newly invented technology which is used for harvesting energy from various mechanical movements such as wind, water wave, and human motion [152]. The large open-circuit voltage (up to several hundred volts) generated by TENG based on triboelectrification and electrostatic induction effect makes it a popular candidate for application in various self-powered wearable devices [153]. In 2017, Gu's group first invented a rotating triboelectric nanogenerator (R-TENG) enhanced electrospun polyimide membrane for air purification. The polyimide nanofibre membrane was positively charged by R-TENG in a constant manner, leading to greatly improved removal efficiency towards PM particles with diameters less than 100 nm (Figure 10(c)) [141]. Using this technology, the same group later developed a self-powered electrostatic adsorption face mask (SEA-FM) from electrospun poly(vinylidene fluoride) membrane equipped with a TENG driven by human respiration (Figure 10(d)) [142]. The SEA-FM exhibited removal efficiency higher than 99.2 wt% towards coarse and fine particulates with a low-pressure drop, and the efficiency towards ultrafine particulates was still as high as 86.9 wt% after continually working for 240 min and a 30-day interval. Very recently, Bai's group presented a washable and reusable multilayer triboelectric air filter which consists of multilayers of nylon and polytetrafluoroethylene fabrics, which can be easily charged through rubbing against each other [154]. A high open-circuit voltage of 190 V can be generated on the surface of the fabrics, leading to a high removal efficiency of 84.7% for PM_0.5_ and 96.0% for PM_2.5_. The removal efficiency is stable under a high humidity environment and displayed no obvious deterioration after five cleaning cycles. Hence, the triboelectric air filters are highly effective and stable with a long service life, paying the way for the fabrication of face masks and other health protection applications.

#### 6.1.3. MOF-Based Filters

**Figure 11:**
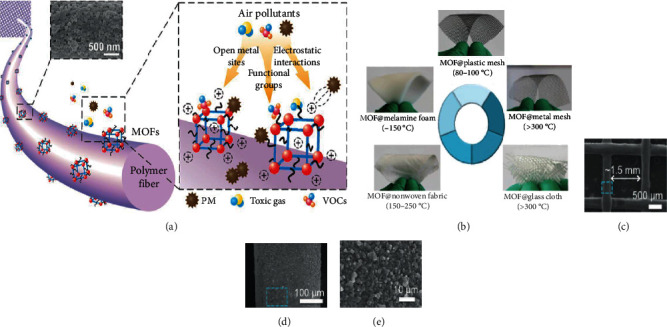
(a) Proposed capture mechanism of the MOF-based filter for air pollutants. Inset is the SEM image of the surface of the MOF/polymer composite fibre. (b) MOF filters based on various flexible substrates produced by a roll-to-roll hot-pressing method. (c–e) SEM images of the ZIF-8@plastic mesh achieved after seven layer-by-layer coating cycles. Reprinted with permission from Ref. [159] and Ref. [156]. Copyright 2016 and 2017, the American Chemical Society and WILEY-VCH.

Metal-organic frameworks (MOFs) are a class of porous crystalline materials that are composed of transition-metal cations and coordinately bonded multidentate organic linkers. With high porosity, tunable pore size, rich functionalities, and good thermal stability, MOFs hold great promise for applications as filtration materials [155]. As MOF crystals are in a light powder form, they are usually grown on porous substrates or embedded in polymer fibrous membranes to form MOF-based filters. Li's group first explored the interactions between MOFs and particulate pollutants via the incorporation of ZIF-8 nanocrystals in electrospun PAN membranes [135]. It was proposed that the particulate pollutants can be captured by the MOF-based filters via three mechanisms: (i) binding to the open metal sites on MOFs, (ii) interacting with the functional groups on MOFs and/or polymers, and (iii) electrostatic interactions with MOF nanocrystals (Figure 11(a)). Due to the unbalanced metal ions and defects on the surface, MOFs can offer positive charge to polarize the PM surface, leading to improved electrostatic adsorption of PM pollutants. The specific surface area of the PAN filter was dramatically improved from 115 to 1024 m^2^ g^−1^ after incorporation of 60 wt% ZIF-8 nanocrystals. The developed ZIF-8@PAN filters exhibited high PM removal efficiencies up to 88.33% and 89.67% for PM_2.5_ and PM_10_, respectively, with an ultralow pressure drop of less than 20 Pa. In addition to PM pollutants, these MOF filters were also found effective in selective capture of SO_2_ when exposed to a stream of SO_2_/N_2_ mixture. Inspired by the promising results, the same group further employed a roll-to-roll hot-pressing method for mass production of MOF-based filters on various commercially available flexible substrates (i.e., plastic mesh, glass cloth, metal mesh, nonwoven fabric, and melamine foam) (Figure 11(b)) [156]. The produced MOF filters demonstrated excellent performance for PM removal under a wide range of working temperatures from 80 to 300°C. The PM removal efficiency of the ZIF-8@plastic mesh (Figures 11(c)–11(e)) was retained >90% after 30 consecutive days. It can be easily washed with tap water and ethanol and reused three times without apparent efficiency loss, which is quite promising for application in residential pollution control. Following Wang's work, Feng et al. designed a hierarchical, multifunctional UiO-66-NH_2_ wrapped CNTs/PTFE filter with a high capture efficiency (99.997%) for ultrafine dust (diameter ∼0.3 *μ*m) and SO_2_ adsorption capacity in dynamic filtration [14]. Koo et al. reported the growth of flowerlike hierarchical 2D assembled MOF on polypropylene microfibres as a washable membrane filter with high PM removal performance (92.5% for PM_2.5_ and 99.5% for PM_10_), low-pressure drop (10.5 Pa at 25 L min^−1^), and superior stability after reuse for 12 cycles [157]. Hao et al. developed electrospun polyimide/ZIF-8 nanofibrous membranes with superior thermal stability (up to 300°C), good transmittance, and excellent mechanical properties for efficient PM_2.5_ capture (up to 96.6% with a 10 wt% ZIF loading), which can be used in harsh conditions such as car exhaust filtration [158].

### 6.2. Improving Filters and Membrane Materials for Microorganism Removal

Though air filters discussed above show excellent capture efficiency for particular matters (PM), microorganisms (or bioaerosols), such as bacteria, viruses, and fungi in the air, adhere to the filter surface, remain viable, and may reproduce within the filter media, which pose a risk of second airborne contamination. Meanwhile, the accumulation of microorganisms in the filter also blocks the filter, leading to reduced ventilation volume and deterioration of the filter [160–162]. Thus, it is highly desirable to develop air filters with antimicrobial properties, especially when the filters are used for respiratory protection, such as masks, and for indoor air purification. Up to now, a wide range of antimicrobial agents, such as natural products, nanoparticles of metal and metal oxide, metal-organic frameworks (MOFs), graphene, and its derivatives, have been investigated to impart air filters with biocidal properties.

#### 6.2.1. Natural Product Extracts

Antimicrobial extracts of natural products have been widely studied as antimicrobial agents for air filters owing to their high antimicrobial activity, low toxicity, low-cost, and gentleness to the environment [163–165]. The microbial toxicity of natural product extracts is generally accredited to the flavonoids they may contain, which kill microbes via the damage of cell membrane function and inhibition of DNA gyrase [160, 162]. Herbal extracts, such as tea tree oils [166], extract of olive [161], extract of *Euscaphis japonica* [164], grapefruit seed extract [165], mangosteen extracts [167], and especially *Sophora flavescens* [168–170], have been sprayed on the surfaces of fibrous polymeric filter for antimicrobial properties, and the herbal extract-coated filter demonstrated good antimicrobial activity. Recently, Sim et al. reported activated carbon (ACF) fibre filters deposited by *Sophora flavescens*, and the as-coated ACF filter exhibited antimicrobial efficiency higher than 90%, with the toluene removing capacity maintained [171]. However, the high antimicrobial activity of these surface-coated filters is often delivered at a high loading of antimicrobial herbal extracts, which would lead to much increased pressure drop [164, 171, 172]. To solve this problem, Choi et al. mixed *Sophora flavescens* with polyvinylpyrrolidone (PVP) solution for electrospinning and thus prepared antimicrobial nanofibrous membrane. Owing to the uniform dispersion of antimicrobial ingredient across the polymeric nanofibres, the fabricated hybrid nanofibrous filter exhibited excellent filtration efficiency (99.99%) and superior antimicrobial activity (99.98%) against *Staphylococcus epidermidis* (*S. epidermidis*), with a low-pressure drop of 3.9% compared with the control at a face air velocity of 1.79 cm/s [173]. However, the durability of natural product extracts is still a concern, when it comes to a real application as the antibacterial activity may be affected by temperature, or degraded due to a natural oxidation process [160, 168, 170]. This can be complemented by nonnatural antimicrobial substances.

#### 6.2.2. Nanoparticles of Metals and Their Compounds

Apart from natural herbal extracts, metals and their compounds have also been extensively studied for their antimicrobial application. Nanoparticles of metal and their compounds have garnered huge attention as a potent antimicrobial agent due to their high surface-to-volume ratios compared with their bulky counterparts [174, 175]. Though each of them has a different mechanism of biocidal action, a generally proposed mechanism includes (i) the disruption of cell membrane metabolism due to the penetration of nanoparticles and/or release of metal ions and (ii) the effect of photocatalytic, as reactive oxygen species (ROS) like hydroxyl (HO^·^) and superoxide radicals (O_2_^-·^) are generated, which induce oxidative stress to microorganisms and cause the ultimate inactivation [174–177].

Nanoparticles of silver (Ag) [178–180], silver compounds (Ag^+^) [181], titanium dioxide (TiO_2_) [182, 183], zinc oxide (ZnO) [184–186], and aluminum and aluminum oxide (Al_2_O_3_) [187] have been incorporated to various filters for antimicrobial properties. A synergistic antimicrobial performance is also revealed via their combination with other biocidal agents, such as carbon nanotubes [178, 180, 181]. Apart from antimicrobial air filters, multifunctional air filters, which simultaneously remove PM, microorganisms, and volatile organic compounds (VOCs), have drawn increasing research attention recently [188, 189]. The integrated multifunctional air filter provides a promising solution to address the high-pressure drop often caused by multiple filters with different functions used in current air filters. Feng et al. designed and fabricated hierarchical Ag/ZnO nanorod-wrapped PTFE nanofibrous membrane with an excellent dynamic antibacterial property of ~100% against *Escherichia coli* (*E. coli*), and a formaldehyde degradation rate of 60%, with slightly increased gas penetration, taking advantages of the antimicrobial properties and photocatalytic abilities of both ZnO and nanosilver [188]. In another study, Zhao et al. reported a multifunctional Ag@MWCNTs Al_2_O_3_ hybrid filter, where Ag@MWCNTs with a hierarchical network-like structure uniformly distributed around the pores of the Al_2_O_3_ filter [189]. The antimicrobial functionality of Ag nanoparticles, as well as their catalytic performance for formaldehyde degradation, were greatly enhanced when loaded on high surface area of CNTs. Owing to the synergistic integration, the Ag@MWCNTs/Al_2_O_3_ hybrid filter demonstrated excellent antimicrobial rate (>98%) against common indoor microorganisms, outstanding degradation of formaldehyde (99.99% at 55°C, and 82.24% at room temperature), and complete retention for particles with sizes ≤ 0.3 *μ*m with a pressure drop of 35.60% compared with the pristine Al_2_O_3_ filter.

Copper nanoparticles are strong microbicides for a broad spectrum of microorganisms [190]. Very recently, they have been demonstrated to be effective against the newly emerged 2019 coronavirus (COVID-19), which is threatening the whole world [175, 190]. Though copper-polymer nanocomposites have been explored for antimicrobial applications, the integration of copper nanoparticles to filters for personal protection or air filtration has not been reported yet. With appropriate technologies to effectively deposit copper nanoparticles onto fibrous filter matrix, more advancement for copper nanoparticles as antimicrobial coating in air filters is expected.

#### 6.2.3. Metal-Organic Frameworks (MOF)

MOFs, as an emerging new class of antimicrobials, are superior compared with metals due to their high surface area, uniform distribution of metal active sites, and adjustable porous structures [191, 192]. There has been rapid progress in recent years on the research of antimicrobial behavior of MOFs, along with the antimicrobial application of MOFs and their composites [191, 193]. The antimicrobial mechanism of MOFs is mainly accredited to the inherent biocidal nature from their metal ions and may also be from the antimicrobial organic ligands [191, 193–195].

**Figure 12:**
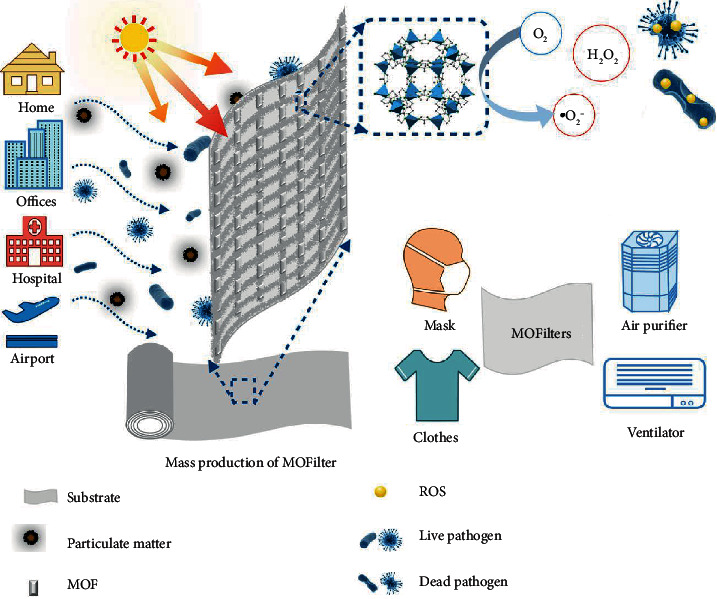
Schematic representation of MOF-based filter (MOFilter) for integrated air cleaning. Reproduced with permission from Ref. [196]. Copyright 2019, Springer Nature.

Ma et al. [195] combined MOFs and cellulose fibre (CF) via simple in situ generation and established multifunctional MOFs@CF air filters. The ZIF-8@CF filter exhibited high filtration efficiency of 98.36% against 0.3 *μ*m particles, high gas adsorption ability, and excellent antibacterial activity against *E. coli* under a pressure drop of 134 Pa. Very recently, inspired by the extremely tunable photocatalytic properties of MOFs, Li et al. exploited their photocatalytic biocidal activities and developed integrated air filters based on the MOFs (Figure 12) [196]. ZIF-8 nanocrystals were integrated to nonwoven fabrics via hot pressing. The established MOFilter achieved 96.8% removal of PM_2.5_ particles with a low-pressure drop (64 Pa) at a flow rate of 0.7 m s^−1^ and bactericidal efficiency higher than 99.99% over 30 min against aerosols containing *E. coli*. The dominant disinfection behavior of ZIF-8 here was ascribed to ROS production from photoelectrons trapped at Zn^+^ centers within ZIF-8 via ligand to metal charge transfer (LMCT), rather than Zn^2+^ releasing. This work sheds light on the photocatalytic biocidal action of MOFs and provides valuable insights for their potential antimicrobial applications in air disinfection.

Though there have been numerous studies on antimicrobial materials and their antimicrobial application, the integration of antimicrobial substances with filters for air purification is still at a preliminary stage, especially for the newly emerging antimicrobial nanoparticles. In view of rising air pollution, and the severe 2019 coronavirus (COVID-19) pandemic, there would be increasing attention on air filters with antimicrobial properties. To achieve high-performance antimicrobial air filters, the choice of highly efficient and biologically safe antimicrobial ingredients, the structural design for good gas permeation, and a simple and economic preparation method are key for their ultimate commercialization to provide protection for public health.

### 6.3. The Development of Masks with Antimicrobial Activity

While face masks can offer some form of protection against airborne and droplet-borne pathogens entering our airway through the mouth and nose, contact with the outer layer of the mask, such as with hands during mask adjustment, removal, or even disposal, can nonetheless result in self-inoculation of pathogenic microbes. A recent study showed that the SARS-CoV-2 virus can remain infectious on the outer layer of the surgical mask even after 6 days [197]. In addition, even accidentally touching a surface for as little as 5 seconds can result in the transfer of some quantity of the infectious microbes to the hands, for example, 32% of influenza A viruses [198]. Disinfection of masks, especially reusable ones, is therefore crucial. Cloth masks may be disinfected by washing with detergents and bleach whereas surgical and N95 masks may be disinfected via UV [199] or heat treatment [116, 200]. Such treatments are nonetheless discrete, and masks are easily contaminated with pathogenic microbes again once in use. Thus, masks with antimicrobial activity that can automatically destroy or inactivate infectious microbes may reduce the risk of contamination.

With increased awareness of epidemics, research on methods to incorporate antimicrobial activity onto masks have intensified. Antimicrobial air filter materials discussed in the previous section (Section 6.2) can be extremely useful in this endeavour. Alternatively, masks may also be treated or coated with antimicrobial agents. Many classes of antimicrobial agents, including metal nanoparticles, organic compounds, and even common household chemicals, have demonstrated antimicrobial activity in masks. Notably, several of these masks are commercially available today; these examples will be highlighted in their respective sections. Some of these masks have also received FDA clearance as a single-use N95 respirator or surgical mask with antimicrobial/antiviral agent (ONT or OUK, respectively) [201, 202], which demonstrate significant antibacterial and antiviral activity in addition to satisfying the basic mask performance standards. Herein, some common antimicrobial agents that may be used to treat masks will be discussed.

#### 6.3.1. Metal-Based Nanoparticles

Metal-based nanoparticles (NPs) are a growing field in the fight against microbes due to their low toxicity towards humans at concentrations effective for pathogen inactivation [203–207]. Due to their broad spectrum of biocidal activity and high potency, most do not induce resistance and are effective against multiresistant bacteria [208, 209]. The two main mechanisms of biocidal activity are as follows: (i) heavy metal ions bind and precipitate thiol (SH) groups in proteins, phosphate (PO_4_^−^) groups in ATP and DNA, and other negatively charged groups in the cell wall/viral envelope, thus causing damage to key microbe functions; (ii) generation of reactive oxygen species (ROS) through changes in redox states or photocatalytic activity, which cause oxidative stress to the microbes.

*(1) Silver Nanoparticles*. Ag and its compounds have broad-spectrum antimicrobial activities and have been widely applied as coatings to medical devices; the high affinity of Ag^+^ to SH is the main mechanism of action [210–212]. There have been several reports of AgNPs conferring antimicrobial properties to masks. One method is to introduce AgNPs onto the materials used to make masks. US Pat. 6979491 disclosed the preparation of antimicrobial yarn by loading the fibrous material with glucose-capped AgNPs. The yarn showed antimicrobial activity against multiple bacteria genus, including *Bacillus*, *Staphylococcus*, *Chlamydia*, *Escherichia*, and *Pseudomonas*, and fungi, such as *Candida albicans* even after dying and 100 times of washing [213]. Anson Nanobiotechnology (Zhuhai) uses this fabric between the electret filter and the inner layer of the mask to produce nanosilver antibacterial surgical masks [99, 214]. Other methods of loading AgNPs onto fabric for mask materials have been described [215–217]. A more facile method could be to coat AgNPs directly onto surgical masks; More et al. showed that masks soaked in a colloidal solution of starch-capped AgNPs possessed antimicrobial activity against both Gram positive (*S. aureus*) and Gram negative (*E. coli*) [218].

*(2) Copper Nanoparticles*. Cu and copper oxide both have potent biocidal properties and have been incorporated into textiles and other products with antimicrobial and antiviral properties [21, 219–223]. The main mechanism of action for CuNPs is the production of ROS during the oxidation of Cu(I). Borkow and coworkers from Cupron Inc. fabricated N95 masks with copper oxide (Cu_2_O and CuO) NPs in both the external and filtration layers [224]. These Cu_x_O-impregnated masks showed not only 99.85% viral filtration efficiencies of human influenza A and avian influenza viruses (similar to control) but also 99.99% reduction in virus titers on the mask surface after 30 minutes. The mask successfully passed European EN 14683:2005 and NIOSH N95 standards. Mask safety was also evaluated: the masks did not cause skin irritation nor poisoning through inhalation or saliva ingestion. A commercially available example is NBC Meshtec's Cufitec® surgical mask, which contains 0.5 wt% CuI in the outer layers [225]. The mask inactivated 99.99% of tested influenza A (H1N1, H2N2, H3N2, H5N1, and H5N9) and B viruses within 5 minutes of exposure and received FDA clearance as an antimicrobial mask (OUK) [226]. Copper (oxide) impregnated fibres have similarly been used in reusable masks. Cupron Inc. produces washable masks made of cotton and patented Cupron® polyester, comprising of Cu_x_O NPs embedded in Rayon fibres [227–229]. Copperline's washable copper knit masks produced from LSK FineTex's patented copper fibres [230, 231] demonstrate >92.3% virus and particulate filtration efficiency, which was increased to 99% with HEPA filter (not reusable), and >99% bacteria reduction after 60 min [232]. Copper Clothing Ltd. produces washable KN99 (FFP3) copper-infused masks; the outermost CuNP fabric was >99% bacteriostatic to *S. aureus* and *P. bacillus* even after 50 washes (patents pending) [233, 234]. Several other copper fibre manufacturers, e.g., CuTEC®, Kuhn Copper Solutions, and CoureTex®, have either teamed up with garment manufacturers or produced copper masks themselves.

*(3) Nanoparticles as Photocatalysts*. Photocatalysts typically inactivate microbes by producing ROS via light-catalysed redox reactions [235–237]. Masks with a surface titanium oxide- (TiO_2_-) apatite layer on the outer nonwoven fabric layer have shown good filtration and photocatalytic activity [238, 239]. Zinc oxide (ZnO) is used in commercially available machine-washable masks with 5 *μ*m particulate filtration developed by Sonovia; the inner polyester fabric of the mask is coated with ZnO NPs using patented ultrasonic cavitation technology, leading to a 98% reduction in surface bacteria *E. coli* and *S. aureus* after 1 h incubation [240, 241]. A recent study showed that even metal-organic frameworks (MOFs) could be used as mask filters. The Zn-imidazolate MOF filter showed >99.99% photocatalytic bactericidal efficiency against *E. coli* aerosol after 30 min and 97% PM removal. When incorporated into a 3-ply mask with nonwoven fabric outer layers, all layers showed almost no measurable level of viable bacteria after 30 min simulated sunlight illumination [196]. Despite the high antimicrobial activity of photocatalyst-impregnated masks, it must be noted that they are only effective when sufficient light energy is applied.

*(4) Multiple Nanoparticle Species*. The wide usage of some metal NPs, especially silver, has led to some bacterial resistance against these agents [242, 243]. Combinations of multiple NP species may be beneficial to the biocidal efficiency. Surgical masks with the outer hydrophobic layer coated with Ag and TiO_2_ NPs showed 100% reduction in viable *E. coli* and *S. aureus* after 48 h incubation while control masks showed a 25% and 50% increase in bacterial counts, respectively; the masks did not cause any skin inflammation or allergy [181]. Argaman Technologies, founded by Gabbay in 2016, produces BioBlockX^TM^ face masks with Respilon® nanofibre filter membrane and 4 patented Argaman Cu-infused layers. The Cu_2_O- and Ag_4_O_4_-impregnated fabrics inhibited 96% of the HIV-1 virus after 30 min and 86% of *E. coli* after 3 h, which was significantly higher than fabrics with only Cu_2_O alone [244, 245]. However, due to the nanofibre filter, the mask should not be laundered.

#### 6.3.2. Common Household Chemicals

*(1) Organic Acids*. Acid-based media, such as citric acid, cause inactivation and aggregation of hemagglutinin (HA) glycoprotein spikes in virus membranes, thus rendering the virus unable to enter cells [246, 247]. The application of citric acid as a coating on the outer layer of face masks was patented as early as 1989 [248] and is currently widely in use. A key example is the GlaxoSmithKline's Actiprotect® N95 respirator, which was the first to receive FDA clearance as an antimicrobial mask (ONT) in 2009 [249], with citric acid coating the outer polyester layer. In addition to passing standardized N95 tests, the Actiprotect® mask inactivated 99.99% of tested influenza A (H1N1, H2N2, H3N2, H5N1, and H5N9) and B viruses within 1 minute of exposure [250].

*(2) Sodium Chloride*. Even coating the PP filtration layer with simple table salt (NaCl) can confer virucidal activity [251]. NaCl-coated filters showed increased filtration efficiency against H1N1 virus aerosols, as well as significantly lowering the virus titers after 5 min incubation; the proposed mechanism is damage to the virus membranes. *In vivo* studies with mice exposed to the H1N1 and H5N1 viruses through NaCl-coated filters showed drastically reduced lung virus titer and increased survival rate compared to the control.

#### 6.3.3. Organic Compounds

*(1) Polyphenols*. Tea polyphenols possess antiviral properties due to the ability of catechin, theaflavin, and their derivatives to damage virus membranes and bind to viral nucleic acids, inhibiting replication in influenza A (H1N1 and H3N2) and B viruses [252, 253]. US patent 5888527 disclosed that dip coating the nonwoven fabric or electret filter of a mask into tea polyphenol extract can inactivate >99% of tested viruses [254]. Catel-Ferreira et al. followed up by grafting catechin onto nonwoven cellulose (Kimberly-Clark® Kimwipes® Lite) using enzyme laccase [255]. While catechin at this concentration did not inhibit *E. coli* bacteria growth, it reduced the surface virus titer of T4D bacteriophage. When used as a filter layer in Kolmi M24001 mask, 99.99% filtration of T4D was observed after 2 h, a 7.5 times improvement over the original filter.

*(2) Cationic Ammonium Compounds*. The biocidal properties of 3-(trimethoxysilylpropyl)dimethyloctadecylammonium chloride and related organosilicon quaternary ammonium chloride- (Si-QAC-) treated surfaces (glass, stone, fibres, metals, and plastic) against bacteria, yeast, algae, and fungi were demonstrated as early as 1971 [256–258]; since then, it has been commercially marketed as Aegis® antimicrobial. Its use as a face mask coating has been disclosed [259, 260] and reusable masks are commercially available from Breathe Healthy® (mask biocidal activity not reported). Cationic ammonium polymers have also been studied: Tiliket et al. coated Kimwipes® with poly(ethyleneimine) (PEI) at pH 6 to increase the protonated cationic form [261]. When used as a filter layer in a Kolmi M24001 mask, 99.999% filtration of T4D bacteriophage was achieved in 1 h. However, live virus was detected on the PEI-coated Kimwipes®, indicating that the virus was captured but not killed.

*(3) Polymers*. The effectiveness of organic acids also led to the development of acidic polymers for mask materials. Dip coating the nonwoven PP filter layer of the mask, or spray coating the polyester outer layer of the mask with solutions of Carbopol® or Gantrez^TM^ S-type polymers, resulted in up to 99.9% reduction in influenza A (H5N1) virus titer after 1 min incubation [262].

#### 6.3.4. 2D Materials

2D materials with a large latera size but atomic-scale thickness are advantageous for antimicrobial applications as their sharp edge has a nanoknife effect that can physically damage the bacterial cell, and some of them also possess outstanding photothermal and photocatalytic properties [263]. Graphene, the superstar of 2D materials, has been most explored as an antimicrobial in various areas [264]. Graphene and its derivatives have also been widely used with other antimicrobial agents, taking advantage of their large surface area, for a synergistic effect to enhance antimicrobial efficacy [265, 266]. Recently, the excellent photothermal properties of graphene in NIR regions have been utilized to increase the surface temperature and thus inactivate microorganisms [267, 268]. Other 2D materials, such as MoS_2_ [269–272] and graphitic carbon nitride (g-C_3_N_4_) [273–275], also show attractive antimicrobial performance, while their potential antimicrobial application in air filtration needs to be further explored.

#### 6.3.5. Combination of Multiple Antimicrobial Classes

Several masks have also integrated more than one class of antimicrobial agents across multiple layers. US patent 7845351 disclosed that treating the outer nonwoven layer with antimicrobial agents comprising polyhexamethylene biguanide, citric acid, and *N*-alkyl polyglycoside, as well as other known antimicrobial agents, can deactivate 99.9% of treated bacteria (MRSA, vancomycin-resistant *E. faecalis*, *M. catarrhalis*, and *K. pneumoniae*), fungus *C. albicans*, and viruses (rhinovirus 1A, influenza A) within 30 minutes of contact [260]. US patent application US20110114095A1 disclosed the use of AgNP-impregnated activated carbon cloth (ACC) as the filtration layer in a face mask [276]; ACC itself showed antiviral activity (93%) against MS-2 coliphage after 6 h incubation, which was enhanced by impregnation of AgNPs (98%). Incorporation of the AgNPs/ACC into a mask resulted in >99.88% virus filtration while having increased air permeability compared to the FFP3 mask. Some FDA-cleared examples of antimicrobial mask include Filigent's BioFriend^TM^ Biomask^TM^ [277–279], cobranded with Medline Curad® Biomask^TM^ (ONT and OUK), as well as Innonix's RespoKare^TM^ mask line (OUK child masks [280]), which both use 2 wt% citric acid on the outermost spun-bound PP layer, as well as Cu(II) and Zn(II) (1.6 wt% each) coordinated to sulfonated Rayon in a second layer before the melt-blown PP filter [281, 282]. Both antiviral masks inactivated 99.99% of tested influenza A (H1N1, H2N2, H3N2, H5N1, and H5N9) and B viruses within 5 minutes. Nexera Medical's SpectraShield^TM^ 9500 masks (ONT) [283] use patented Ag-Cu zeolite [284, 285] (Agion®, Sciessen LLC) in the outer PET fibre layer [286] (Fosshield®, Foss Manufacturing); it kills 99.99% of tested bacteria (*S. pyogenes*, MRSA, and *H. influenzae*) and can also inactivate SARS, influenza, and filovirus. It has been tested for continuous use for up to 8 hours. Similar technology has also been described elsewhere [287].

In summary, multiple types of biocidal agents have been incorporated into masks, giving them the added ability to kill pathogenic microbes while not adversely affecting their basic performance. To date, many masks, some including more than one type of antimicrobial agent, are commercially available as summarised in Table 3. However, it must be noted that mask antimicrobial activities have only been studied under strict laboratory conditions; the actual performance of the masks during day-to-day usage may vary. For example, the time required to achieve high biocidal activity may be dependent on the amount of light, especially for photocatalytic biocidal agents, humidity, or airflow. The performance of reusable masks after repeated washing, especially after laundering with surfactants and at high temperatures, may also differ from the laboratory tests. Hence, while antimicrobial masks can offer additional protection against microbes, basic hygiene practices such as not touching the mask surface and washing of hands should still be observed.

### 6.4. Masks with Other Functional Properties

Increasingly, wearing of face masks is becoming the new norm in our lives. In an attempt to curb the spread of the virus, more than 50 countries have made face masks mandatory in public spaces during the COVID-19 pandemic, such as China, Singapore, Spain, and France [290]. In other places, medical experts highly encourage the use of face masks for the protection of the community and oneself against viral transmission. As international travel bans gradually lift, airlines require passengers to don masks at all times. Experts are anticipating a prolonged period of such measures as the world battles the disease. Even when we leave the shadows of the COVID-19 pandemic, we prepare and anticipate future health crises, especially those of a respiratory and infectious nature. We could perhaps no longer treat mask-wearing as a temporary solution but to adapt to having face masks as part of our staple of accessories. With that comes an array of peripheral issues and problems to address, not necessarily medical in nature, in the “new norm.” The following paragraphs aim to highlight several of these. To note also is the matter of cost—as a temporary transient accessory or a surgical PPE, currently mask manufacturing tends to aim at driving the cost down, at producing cheap disposable units. With it becoming a staple, people may be convinced to invest more in sophisticated, multifunctional reusable variants, and cost-per-wear will help justify the addition of these attributes.

#### 6.4.1. Super Hydrophobicity

In addition to their antimicrobial activities, functionalized graphene and graphene-based composites have been reported to confer superhydrophobicity onto material surfaces. Very recently, Zhong et al. [232] established the deposition of few-layer graphene onto commercial nonwoven masks via dual-mode laser-induced forward transfer. The graphene-deposited mask exhibited outstanding superhydrophobic and photothermal performance. The superhydrophobic surface of a graphene-coated mask can effectively repel the incoming aqueous droplets, while the as-coated mask surface can go up to 80°C under sunlight illumination to achieve self-sterilization. More impressively, the roll-to-roll laser production system can be integrated with current roll-to-roll surgical mask production lines, and the cost of raw materials is low, which makes the technology promising for commercial applications. Furthermore, the graphene-coated masks can be further recycled for solar-driven desalination.

#### 6.4.2. Transparent Quality

There are three main categories of social needs that require a transparent quality to our everyday face mask: the hearing-impaired, the digital face-recognition technology, and the human-facing industry. There are an estimated 466 million in the world suffering from deafness or hearing loss [291], who heavily depend on lip-reading for communication. With the loss of partial face visibility, a substantial population of the hearing-impaired will be adversely affected in speech perception. The world is also increasingly dependent on digital facial feature recognition technology—be it in airport/border control, CCTV monitoring (for surveillance and security), or unlocking our mobile phones. Face recognition algorithms have not been optimised for the mask-wearing era, and the lack of which directly threatens societal security. Lastly, for the human-facing industry or population, reading of facial expressions can be crucial [292, 293], for instance, interpreters and translators, caretakers for people with illnesses, confusion and anxiety, customer-facing staff (including medical staff where medical miscommunication may occur), interacting with people who speak a different language, or the elderly and the young. Mehrabian and Ferris have popularised the importance of nonverbal communication: 55% of communication is visual [294]. Reading one's full face provides nontrivial cues to accessing another fellow human being.

As tech companies attempt to resolve the second category of digital recognition using enhanced algorithms (such as the Israeli Corsight and the Chinese Hanvon) for covered faces [295, 296], we envisage a transparent mask as a straight-forward solution to the aforementioned needs. The challenge is threefold for a transparent mask material—it needs to be nonpermeable to liquids and fluids, it should provide two-way protection against transmission (of viral shedding), and it has to be breathable for human wear. There have been numerous transparent face mask patents in the market [297–301], for instance, one from 2010 that in particular highlights a disposable transparent antimicrobial face mask [259]. For medical purposes, it consists of highly porous transparent film, with nonporous microvented laminae to provide controlled gas permeation (but liquid-proof). The transparent panel is said to be made from thermoplastic films such as polyethylene terephthalate (PET) and polyvinylidene fluoride (PVDF), with perforations and pleats incorporated. The antimicrobial used here is a Si-QAC biocide that provides mechanical contact kill, to prevent chemical leaching from conventional antimicrobials. It forms a durable coating on porous textiles and films, by initially bonding to the target surface (of the mask), and thereafter copolymerising between the target surface and itself, resulting in no “dislodgeable residue, odour, leaching, off-gassing, migration, or diffusion of the molecule.”

Another interesting patent from 1984 points to a “transparent, odour-free face mask” [302]. The inventor J.H. Steinberg proposes using invisible or transparent materials and antibiotic/antiodour solutions with a transparent resin. The bactericides and deodorant solutions are either embedded in the resin or coated on the resin sheet. This thin flexible foraminous resin or sheet would then be shaped into a cup/mask form.

More recently, commercial product ClearMask claims to be the first fully transparent face mask, patent-pending at the time of writing [300], for ease of connecting and communicating with people, focusing on a human-centric experience. The patent application appears to describe a design where the transparent plastic piece can also act as an “impermeable barrier for air and/or other particulates,” and breathability is afforded by airflow from the side of the mask (but preventing airflow from/to top and bottom of the mask) when worn. Other products have also appeared on the market, for instance, Shieldofglory's Transparent Hygenic Masks, covered under their 2013 patent [303], offering reusable (and refillable) solutions for preventing saliva or bacteria; however, their antiviral ability is not known.

#### 6.4.3. Comfort, Convenience, and Cleaning

With prolonged wearing of masks being necessary, such as for hours on flights and enclosed or confined spaces, offices, and workplaces, the inconvenience and discomfort, especially for children and the elderly, are amplified by current mask designs. It usually boils down to a trade-off between comfort/breathability and filtration power. For instance, Konda et al. surveyed common fabrics used in cloth masks and evaluated their aerosol filtration efficiency for particulate sizes of 10 nm to 10 *μ*m [95]. The studies found hybrid fabrics are better at filtering out particles, likely due to the combined mechanical and electrostatic filtration ability of the materials. Nonetheless, a maximum of 80% filtration efficiency for particles under 300 nm has been observed in that study, compared to 95% in N95 masks. Cloth or homemade masks also tend to fit less well, further reducing their filtration efficiencies. Other efficacy studies indicate a similar trend of commercial surgical masks being a preferred viral barrier to homemade masks [12, 17, 304].

Materials and designs promising of better breathability, durability, and comfort have been proposed and invented for mask use. Several suggest the use of microporous membranes, or films with interconnected pores, for ventilation and breathability. Increasing the space between the mask and nose/lip area and enhancing the softness/flexibility of the material, ear loops, and the weight of material can all contribute towards the overall comfort [305–311]. One interesting feature, for instance, is encapsulating discontinuous patterns of a phase change material aimed at cooling the microclimate of the inside of a mask, by ~1-7°C, or by >30 J [312]. The invention gives an example of microencapsulated paraffin wax; the transition temperature of the phase change material can be chosen in the range of 25-29°C. Heat from the person's exhalation is absorbed by the material, which melts in the process, henceforth reducing the rise in temperature in the small space. The material resolidifies as it releases the energy to the ambient air during inhalation. The process can then be cycled. Such material is coated in a discontinuous pattern so as not to reduce coverage of the liquid-resistant barrier material. A cooling function is especially critical in a tropical climate [313, 314]. Singapore's Innosparks has produced an “AIR+ mask,” incorporating a microfan onto an N95 face mask for the said purpose [315].

The “LMP S2” mask replaces the N95 construction for silicone in a streamlined design, claiming to ensure an air-tight and more conformable fit, and is softer on one's skin. The reusable mask comes with removable filters. Another design by BDCI involves a simple 3D-printed skeleton to reinforce the mask shape firmly, preventing negative air pressure from collapsing the mask cup during inhalation, hence improving the ease of breathing [316].

The ability to self-clean is certainly desirable. Disposable masks are usually not environmentally friendly and require constant manufacturing and purchasing. Having to wash reusable masks daily (or more) would increasingly be out of sync with the population's lifestyle. Apart from the aforementioned antimicrobial properties, a few self-cleaning or self-sterilizing approaches are available for repeated prolonged or repeated use of masks. Stanford et al. demonstrate self-sterilizing laser-induced graphene (LIG) in air filters [266]. The LIG has a high surface area and traps microbes whose proliferation is inhibited on the graphene. Cycles of Joule heating then kills the microorganisms and pathogens, and the high thermal stability of LIG allows it to be reused. Arnusch et al. took this LIG and LIG-composite nanotechnology further, extensively applied to wastewater treatment and water filters, using an applied electrical potential to be bactericidal [317–320]. Since the onset of the COVID-19 pandemic, Arnusch et al. are trying to commercialize the technology onto a face mask (“Guardian G-Volt”), developed by the LIGC Applications company. The LIG self-disinfectant system can be plugged into a portable battery or a home-dock via a USB port, and an applied electrical potential “fully sterilizes” the mask for safe reuse [321]. Zhong et al. in a separate effort, developed a dual-mode laser-induced forward transfer method for depositing graphene layers onto low-melting temperature surgical masks. The graphene coating functions as an aqueous-resistant layer on the mask surface. The surface temperature also heats up easily and quickly to >80°C under sunlight, naturally sterilizing itself, pushing towards reusable and recyclable graphene masks [232].

One other common approach is the use of ultraviolet (UV) light. One early demonstration by inventor Ricci showed a germicidal mask [322], where the air breathed in by the user has been exposed to inbuilt UV radiation, killing pathogens and viruses. Likewise, the user's exhaled air can also be disinfected before being released to the ambience. More recently, the leading wearable tech company Huami's new product in development, Aeri mask, has an inbuilt UV light that can disinfect the mask filters within 10 min when connected to a power supply via USB cables [323]. When realised, this would make efficient reusable face masks.

#### 6.4.4. Good-to-Haves: Function, Fashion, and Future

We live in a constantly image-conscious world of consumerism, social media, and complicated human psyche. As we transit into the new postpandemic age, we cannot ignore the social and psychological aspects of donning additional apparel. Aesthetics, trends, and fashion are important factors in everyday living, as we already see reusable fabric masks sporting various patterns and designs. Multiple functions, way beyond the essential medical needs and convenience, would be desired. Masks now provide a ready platform to incorporate gadgets, electronics, good-to-haves, and a canvas for novel ideas. As our postpandemic lifestyle evolves, it is appropriate to consider the fashion, function, and future of masking up.

It is inspiring to take creative design approaches into consideration when evaluating the next-generation protective masks. Yanko Design has gathered many innovative technology ideas along this line. In one, VYZR Technologies create a product BioVYZR, a purified-air bubble around one's head worn like a vest-strap, with all-around antifogging visibility, safe space, filtered air as an N95 mask, and positive air pressure for easy breathing. The setup when charged can run for 8-12 hours and is said to be more spacious and comfortable [324]. Another designer Joe Doucet takes on a fashionable stand with a sleek face mask-shield-visor all in one [325].

In terms of functionality, smart mask designs move towards air quality sensors/monitors, biochemical or physical sensors, health trackers, and even bone conduction headphones, etc.

*(1) Communication Tools*. Several products on the market now have incorporated bone conduction headphones/earphones and even microphones onto the mask design [316, 326, 327], currently aimed at the outdoor sports community. Such features also help to enhance communication between the everyday mask-wearing population, when our voices are muffled by the mask materials.

*(2) Breath Sensors*. There are several reports of nanosensors embedded in surgical masks for monitoring human breathing rate, most of which use materials which produce electrical outputs in response to humidity during human breathing [328–330]. The Laboratory for Embedded and Programmable Systems (UC Davis) has also embedded oxygen, carbon dioxide, and flow sensor into an elevation training mask (Training Mask 2.0®) [331]. Abnormal breath patterns can indicate poor health or even lung disease [273, 331].

*(3) Usage and Air Quality Sensor*. Xiaomi Inc. has patented a smart mask which can record wearing time, respiratory rate, and download pollution level data [332]. From these data, the mask can calculate its pollutant absorption quantity and signal to the user when the mask should be replaced; the mask is expected to be commercially available soon. Xiaomi's current product AirPOP mask, targeted at protection against airborne pollution, also has its app-accessible integrated sensor chip clipped onto the mask surface.

*(4) Self-Powered Masks*. Zhou et. al. developed a bifunctional mask with an electret filter layer capable of also acting as a nanogenerator, breath, and even mask life sensor [333]. Using negatively charged electrospun PEI as the nonwoven electret, 99.6% particle (0.3 *μ*m) removal efficiency was achieved. The PEI electret can be loosely sandwiched between 2 iron (Fe) net electrodes to form a nanogenerator and incorporated into a commercial mask; during exhalation, the airflow causes movement of the electret between the Fe electrodes, generating alternating electricity to power a small LCD screen, which can display the measured breathing rate. As the surface charge of the PEI electret decreases, removal efficiency and power generated will decrease; thus, when breathing can no longer power the LCD, the mask should be replaced. The mask has been tested for 40 h of continuous use.

*(5) Microbe Detectors*. Face mask sampling has been used by Williams and coworkers to detect *M. tuberculosis* in breath [334]. A gelatine membrane sampling matrix in the mask collects breath and sputum from the user over 8 h, which is then sent for laboratory PCR testing. This method is noninvasive and enabled early detection of tuberculosis in people whose tuberculous burden is too low to be detected by conventional sputum tests. The team is now working on developing polyvinyl alcohol test strips for mask sampling for SARS-CoV-2 virus to detect COVID-19 [335]. Another team of researchers led by Collins developed a paper-based colorimetric sensor for the detection of Zika virus RNA [121]. The time for Zika diagnosis was shortened to less than 3 h with this sensor. The scientists are now designing a face mask which can detect SARS-CoV-2 virus and produce a fluorescent output [336].

As inspiring as it appears, the market is still waiting for a truly multifunctional multipurpose product, or one that can be tailored to one's needs. The Chinese tech company Huami has recently announced their new design of a transparent N95-like mask (the “Aeri mask”) that incorporates multiple functions such as unlocking phones with facial recognition while wearing it, self-cleaning, removable air filters (with each filter lasting 1.5 months), antifogging, and in-built fan for breathable comfort. The product is said to sport a modular design, therefore allowing for customizable features according to one's needs. Prototyping is in progress at the time of writing [323].

## 7. Future Perspective

Following the World Health Organization's (WHO) first recommendation of wearing face masks in the general public in early April 2020 as the coronavirus spread globally, the demand for face masks escalated. This generated a demand for raw materials and environmental impact. A recent study from UCL suggests that if every person in the UK uses one single-use mask each day for a year, 66,000 tonnes of contaminated plastic waste would be generated, without counting the waste from packaging [337]. Assuming the same disposal rate for every affected country, the medical waste generated at a global scale is going to be substantial, with a negative impact on our ecosystem and human health [338].

Apart from physical waste, greenhouse gas (GHG) emission across the mask life cycle is another concern. GHG is emitted at every stage of the face mask life cycle, from the production of polymer resin, nonwoven sheet conversion, face mask assembly, and transportation to the end of life (EoL) treatments by incineration or landfill. It was reported that 1 kg fossil fuel-based plastics could emit 4.1 kg of CO_2_-equivalent (CO_2_e) over the total life cycle on average in 2015, among which over 60%, about 2.7 kg CO_2_e, was released from the polymer resin production stage [339]. Based on the UCL projection of 66,000 tonnes of mask waste generated each year in the UK at the current high mask disposable rate, 178,200 tonnes of GHG could be released into the environment per year solely from the resin production stage. The subsequent energy-intensive manufacturing process such as melt-blown, transportation, and incineration is expected to further increase the carbon footprint of mask to a large extend. To have a clear picture of the exact impact being imposed by face masks, comprehensive life cycle assessment (LCA), a well-adopted methodology for analysing the environmental impact of an industrial system from cradle to grave, should be used. There is no doubt that the sudden increase in mask manufacturing and usage will cast more pressure to the already alarming global environmental issues originated from plastic products.

On the one hand, more masks are needed for reducing the risk of the virus spreading (part of the preparations for economic reopening). On the other hand, reducing mask production, usage, and disposal is preferred for environmental reasons. Facing such a dilemma, tackling both challenges may need synergistic efforts from policymakers, industry players, researchers, and the general public. These efforts include strategies to reuse disposable masks, a search for alternative mask material with low GHG emission, etc. Amid the crisis, we are seeing promising initiatives from the industrial and research communities.

The reuse of single-use face masks provides a straightforward way to reduce the disposal rate. In this regard, appropriate disinfection processes for safe and frequent reuse of masks without composing the mask filtration efficiency need to be determined. This has been discussed in detail in the previous section (Section 5). Besides recycling commercial masks, DIY reusable masks also help to alleviate the supply shortage, with possible environmental benefits. As discussed in Section 4, with the right choice of materials, appropriate design, and assembly, a DIY reusable mask may function as well as commercially manufactured surgical ones. Using easily available household materials such as reusable nonwoven bag, dried hypoallergenic wet wipe, and a thin cotton cloth as the outer hydrophobic, middle filtering, and inner adsorbent layers, respectively, researchers from A∗STAR, Singapore, successfully designed a DIY mask with essential properties comparable to a surgical mask [340]. As the performance of DIY masks depends on the materials used and how it is they are assembled, the public should not have the misconception that DIY masks can serve as a surgical mask alternative.

A changeable filter layer that can be fitted inside the masks prevails as a viable option in improving the performance of a self-improvised mask. The replaceable layer of fibrous material filters out viruses and other pathogens and allows the mask to be washed and reused. The team led by Jung at KAIST developed a versatile nanofibre filter that can be fitted inside a mask using an “Insulated Block Electrospinning (IBE)” [341]. The changeable filter endows the various grass-root production of masks over the past few months with up-to-standard filtration performance.

In addition to the essential filtering function, incorporating multifunctionality into mask design or mask materials opens up opportunities for masks with advanced features. “Self-sanitising” and “self-cleaning” are among the new terminologies being used in the future “greener” mask. Some of these innovative efforts have been described in Section 6.4.

Spurred by the outbreak of COVID-19, many companies are transitioning to mask production to meet the vast demand, such as global gaming hardware manufacturing company Razer Inc. Yet, this ambitious effort has run into a bottleneck, the deficit of melt-blown fabric. Putting supply availability aside, the life cycle GHG emission level of the raw materials poses another set of challenges. A remedy to these problems could be found in the development of “green” substitute products based on the polymer with lower GHG emission level or bio-based materials produced from plants.

For instance, Krucińska et al. earlier developed biodegradable particle-filtering respiratory half-masks from nonwoven poly(lactic acid) (PLA) which met the requirements of the low (FFP1) and medium (FFP2) protection classes against harmful aerosols [342]. Zhang et al. also reported the development of biodegradable electrospun poly(l-lactic acid) (PLLA) polymer nanofibres that can significantly improve the removal of PM_2.5_ particulates via the generation of electrostatic charges. The filter membrane exhibited a high efficiency of 99.3% and compared to a 3M respirator, still exhibits a 15% improvement in quality factor after 6 hours of filtration time [343]. Guzdemir and Ogale recently reported producing fibres with reduced content of PP for disposable fabrics by incorporating bio-based renewable material, soy flour, where thin fibres with a diameter under 60 *μ*m can be successfully melt-spun [344]. It was found that the presence of soy particulates on fibre surface enhanced its water absorption and colourability properties while retaining the feel of natural fibres. More recently, researchers from the BioProducts Institute at the University of British Columbia have developed a fully compostable and biodegradable medical N95 mask, named Can-Mask, using wood fibres from sources such as pine, spruce, cedar, and other softwoods [345].

Although a large range of thermoplastic resins can be processed by spun-bond and melt-blown technology, an essential part of the mask structure, PP remains the major resin being used for its ease in processing, especially in terms of viscosity control. Hence, proper control of the rheological behavior using modifiers will assist in the production of non-PP melt-blown fabric that meets the requirement for medical applications. Natural materials such as cellulose, cotton, and commercial resins such as Bioplast could become potential candidates after issues regarding their poor thermal stability have been addressed.

Recent efforts have demonstrated the potential of reusable mask development enabled by material innovation and technology advancement in addressing the mask shortage while reducing the GHG emissions and negative environmental impact. However, continuous efforts are needed to ensure feasible developments can be transit to existing manufacturing facilities. Also, there are more scientific opportunities to develop novel and environmentally friendly mask materials with functions of interests such as self-sanitising and degradable materials and to develop a low energy consumption technique or process for a nonwoven fibre that could replace a carbon-intensive melt-blown process in the near future. This does not undermine the role of a single-use mask as an immediate measure to protect those at high risk of infection healthcare professionals. Till date, COVID-19 is still on the rise worldwide, the use of mask may become a norm and the demand will remain high. Hence, continuous efforts are required for a closely integrated experimental and theoretical investigation aiming to progress upon the current state of understanding and perpetuate the development of innovative solutions for the mask crisis amid the pandemic.

## 8. Conclusion

The COVID-19 pandemic has forced the global population to adopt new ways of living, including the wearing of masks as a new norm. It has even accelerated R&D efforts in mask materials and design to offer better protection for users against airborne pollutants and pathogens. This review therefore provides a holistic summary of the A to Z of face masks, to give readers a broad-view understanding of masks from the perspective of public health to the domains of material development. The importance of mask-wearing in preventing the spread of airborne and droplet-borne infections was discussed early in this review. Thereafter, the protection mechanism, production, and performance testing of commercial masks were described. We then explored the effectiveness of DIY homemade masks as an alternative to commercial masks. To overcome the issue of mask shortage, methods to decontaminate used masks were introduced and elaborated. The review then discussed research advances in the development of materials with improved filtering capacity and antimicrobial activity. This was followed by R&D efforts in the engineering of multifunctional masks with properties such as antimicrobial activity, hydrophobicity, transparent see-through surfaces, sensing-cleaning, self-powered, and even sensing and detection capabilities. Finally, the environmental implications of widespread mask-wearing and increased mask production were deliberated upon as efforts towards finding more sustainable solutions to support long-term mask-wearing, even after the end of the pandemic, were explored. As mentioned in the very beginning, the fight against any infectious diseases requires efforts and solutions in prevention, detection, diagnosis, and treatment. The wearing of masks therefore serves as a key strategy towards airborne disease prevention that cannot be easily substituted.

## Figures and Tables

**Table 1:** Comparison of commercial mask types, performance, and use cases.

Types	BFE@ 3.0 *μ*m	PFE@ 0.1 *μ*m	Fluid resistance (mmHg)	Delta P mm H_2_O/cm^2^	Flame spread	Use case
N95	≥99.9%	99.9%	160	>5.0	Class 1^a^	Used when treating patients with diseases such as TB, measles, or influenza.
ASTM level 3	≥98%	≥98%	160	<5.0	Class 1^a^	Used for procedures where heavy levels of aerosols, spray, and/or fluids are produced.
ASTM level 2	≥98%	≥98%	120	<5.0	Class 1^a^	Used for procedures where light to moderate levels of aerosols, spray, and/or fluids are produced.
ASTM level 1	≥95%	≥95%	80	<4.0	Class 1^a^	Used for procedures where low levels of aerosols, spray, and/or fluids are produced.
Utility mask (low performance)	N/A	N/A	N/A	N/A	N/A	Used as a simple physical barrier for dry, short procedures without fluid, spray, or aerosols.
Utility mask (minimum performance)	N/A	N/A	N/A	N/A	N/A	Used as a simple physical barrier for dry, short procedures without fluid, spray, or aerosols.

^a^Class 1 flammability requires an average burn time of ≥3.5 s [77].

**Table 2:** The filtration efficiency and pressure drop across materials with two different microorganisms. Reproduced with permission from Ref. [12]. Copyright 2013, Cambridge University Press.

Material	*B. atrophaeus*		Bacteriophage MS2		Pressure drop across fabric	
	Mean % filtration efficiency	SD	Mean % filtration efficiency	SD	Mean	SD
100% cotton T-Shirt	69.42 (70.66)	10.53 (6.83)	50.85	16.81	4.29 (5.13)	0.07 (0.57)
Scarf	62.30	4.44	48.87	19.77	4.36	0.19
Tea towel	83.24 (96.71)	7.81 (8.73)	72.46	22.60	7.23 (12.10)	0.96 (0.17)
Pillowcase	61.28 (62.38)	4.91 (8.73)	57.13	10.55	3.88 (5.50)	0.03 (0.26)
Antimicrobial pillowcase	65.62	7.64	68.90	7.44	6.11	0.35
Surgical mask	96.35	0.68	89.52	2.65	5.23	0.15
Vacuum cleaner bag	94.35	0.74	85.95	1.55	10.18	0.32
Cotton mix	74.60	11.17	70.24	0.08	6.18	0.48
Linen	60.00	11.18	61.67	2.41	4.50	0.19
Silk	58.00	2.75	54.32	29.49	4.57	0.31

**Table 3:** Properties of commercially available antimicrobial masks.

Mask brand	Classification^a^	Antimicrobial in outer layer	Antimicrobial in inner layer	Mask proven biocidal against	Ref.
Nexera Medical SpectraShield^TM^	N95 (ONT)	Ag-Cu zeolite	X	Bacteria; virus	[283–286]
GlaxoSmithKline Actiprotect®	N95 (ONT)	Citric acid	X	Virus	[249, 250]
Filigent BioFriend^TM^ Biomask^TM^	N95 (ONT) Surgical (OUK)	Citric acid	Cu NPs Zn NPs	Virus	[277–279, 281, 282]
Innonix RespoKare^TM^	Surgical (OUK)	Citric acid	Cu NPs Zn NPs	Virus	[280–282]
NBC Meshtec Cufitec®	Surgical (OUK)	CuI NPs	X	Virus	[225, 226]
Anson Nano Silver	Surgical	X	AgNPs	Bacteria; fungi	[214, 288]
Copper Clothing	Washable KN99 (FFP3)	Cu_x_O NPs	X	Bacteria; virus; fungi^b^	[233, 234]
Cupron Inc.	Washable	Cu_x_O NPs	X	Bacteria; virus; fungi ^b^	[227–229]
Copperline	Washable^c^	Cu_x_O NPs	X	Bacteria	[230, 231, 289]
Argaman BioBlockX^TM^	Reusable^d^	Cu_2_O NPs Ag_4_O_4_ NPs	Cu_2_O NPs Ag_4_O_4_ NPs	Bacteria; virus	[244, 245]
Sonovia Sonomask^TM^	Washable	X	ZnO NPs	Bacteria	[240, 241]

^a^Medical and/or FDA classification. OUK = FDA clearance as a surgical mask with antimicrobial/antiviral agent; ONT = FDA clearance as an N95 mask with antimicrobial/antiviral agent. Washable/reusable masks do not require any certification. ^b^Antimicrobial tests were performed only on fabric but not directly on the masks. ^c^Single HEPA filter provided with the mask is not washable or reusable. ^d^Reusable but not recommended to be laundered.
